# Author Correction: WWP2 regulates pathological cardiac fibrosis by modulating SMAD2 signaling

**DOI:** 10.1038/s41467-019-12060-5

**Published:** 2019-09-09

**Authors:** Huimei Chen, Aida Moreno-Moral, Francesco Pesce, Nithya Devapragash, Massimiliano Mancini, Ee Ling Heng, Maxime Rotival, Prashant K. Srivastava, Nathan Harmston, Kirill Shkura, Owen J. L. Rackham, Wei-Ping Yu, Xi-Ming Sun, Nicole Gui Zhen Tee, Elisabeth Li Sa Tan, Paul J. R. Barton, Leanne E. Felkin, Enrique Lara-Pezzi, Gianni Angelini, Cristina Beltrami, Michal Pravenec, Sebastian Schafer, Leonardo Bottolo, Norbert Hubner, Costanza Emanueli, Stuart A. Cook, Enrico Petretto

**Affiliations:** 1 Programme in Cardiovascular and Metabolic Disorders, Duke-NUS Medical School, Singapore, 169857 Republic of Singapore; 2 Department of Emergency and Organ Transplantation (DETO), University of Bari, 70124 Bari, Italy; 3 SOC di Anatomia Patologica, Ospedale San Giovanni di Dio, 50123 Florence, Italy; 4 National Heart and Lung Institute, Imperial College London, London, SW7 2AZ UK; 5 Unit of Human Evolutionary Genetics, Institute Pasteur, 75015 Paris, France; 6 Division of Brain Sciences, Imperial College Faculty of Medicine, London, W12 0NN UK; 7 Animal Gene Editing Laboratory, BRC, A*STAR20 Biopolis Way, Singapore, 138668 Republic of Singapore; 8 Institute of Molecular and Cell Biology, A*STAR, 61 Biopolis Drive, Singapore, 138673 Republic of Singapore; 9 MRC London Institute of Medical Sciences (LMC), Imperial College, London, W12 0NN UK; 10 National Heart Centre Singapore, Singapore, 169609 Republic of Singapore; 11 Cardiovascular Research Centre, Royal Brompton and Harefield NHS Trust, London, SW3 6NP UK; 12 Centro Nacional de Investigaciones Cardiovasculares – CNIC, 28029 Madrid, Spain; 13 Bristol Heart Institute, Bristol Medical School, University of Bristol, Bristol, BS2 89HW UK; 14 Institute of Physiology, Czech Academy of Sciences, 142 00, Praha 4, Czech Republic; 15 Department of Medical Genetics, University of Cambridge, Cambridge, CB2 0QQ UK; 16 The Alan Turing Institute, London, NW1 2DB UK; 17 MRC Biostatistics Unit, University of Cambridge, Cambridge, CB2 0SR UK; 18 Cardiovascular and Metabolic Sciences, Max Delbrück Center for Molecular Medicine in the Helmholtz Association (MDC), 13125 Berlin, Germany; 19 DZHK (German Centre for Cardiovascular Research), Partner Site Berlin, 13347 Berlin, Germany; 20 Charité-Universitätsmedizin, 10117 Berlin, Germany; 21 Berlin Institute of Health (BIH), 10178 Berlin, Germany

**Keywords:** Ubiquitin ligases, Cardiomyopathies

Correction to: *Nature Communications*; 10.1038/s41467-019-11551-9, Article published online 09 August 2019.

The original version of this Article contained an error in the spelling of the author Massimiliano Mancini, which was incorrectly given as Massimilano Mancini. This has now been corrected in both the PDF and HTML versions of the Article.

