# Supplementary material for: WWP2 regulates pathological cardiac fibrosis by modulating SMAD2 signaling
Source: Nat Commun. 2019 Aug 9;10:3616. doi: 10.1038/s41467-019-11551-9 (PMC6689010; doi:10.1038/s41467-019-11551-9)
Supplement: Supplementary file 1 — Supplementary information [file 41467_2019_11551_MOESM1_ESM.docx]

**SUPPLEMENTARY INFORMATION**

**WWP2 regulates pathological cardiac fibrosis by modulating SMAD2 signaling**

Huimei Chen^1,^*****, Aida Moreno-Moral^1,^*****, Francesco Pesce^2^, Nithya Devapragash^1^, Massimilano Mancini^3^, Ee Ling Heng^4^, Maxime Rotival^5^, Prashant K. Srivastava^6^, Nathan Harmston^1^, Kirill Shkura^6^, Owen J. Rackham^1^, Wei-Ping Yu^7,8^, Xi-Ming Sun^9^, Nicole Gui Zhen Tee^10^, Elisabeth Tan^1^, Paul JR Barton^4,11^, Leanne E Felkin^4,11^, Enrique Lara-Pezzi^12^, Gianni Angelini^4,13^, Cristina Beltrami^4^, Michal Pravenec^14^, Sebastian Schafer^1,10^, Leonardo Bottolo^15,16,17^, Norbert Hubner^18,19,20,21^, Costanza Emanueli^4,11^, Stuart A Cook^1,9,10^ and Enrico Petretto^1,9,¶^

**Authors affiliations:** ^1^Programme in Cardiovascular and Metabolic Disorders, Duke-NUS Medical School, 8 College Road, 169857 Singapore, Republic of Singapore; ^2^Department of Emergency and Organ Transplantation (DETO) University of Bari, 70124 Bari, Italy; ^3^SOC di Anatomia Patologica, Ospedale San Giovanni di Dio, 50123 Florence, Italy; ^4^National Heart and Lung Institute, Imperial College London, London SW7 2AZ, UK; ^5^Institute Pasteur, Unit of Human Evolutionary Genetics, Paris 75015, France; ^6^Division of Brain Sciences, Imperial College Faculty of Medicine, London W12 0NN, UK; ^7^Animal Gene Editing Laboratory, BRC, A*STAR, 20 Biopolis Way, 138668 Singapore, Republic of Singapore; ^8^Institute of Molecular and Cell Biology, A*STAR, 61 Biopolis Drive, 138673 Singapore, Republic of Singapore; ^9^MRC London Institute of Medical Sciences (LMC), Imperial College Faculty of Medicine, London W12 0NN, UK; ^10^National Heart Centre Singapore, Singapore 169609, Republic of Singapore; ^11^Cardiovascular Research Centre, Royal Brompton and Harefield NHS Trust, London SW3 6NP, UK; ^12^Centro Nacional de Investigaciones Cardiovasculares – CNIC, 3 Melchor Fernandez Almagro, 28029 Madrid, Spain; ^13^Bristol Heart Institute, Bristol Medical School, University of Bristol, Bristol BS2 89HW, UK ^14^Institute of Physiology, Czech Academy of Sciences, Vídeňská 1083, 142 00 Praha 4, Czech Republic; ^15^Department of Medical Genetics, University of Cambridge, Cambridge, CB2 0QQ, UK; ^16^The Alan Turing Institute, London NW1 2DB, UK; ^17^MRC Biostatistics Unit, University of Cambridge, Cambridge CB2 0SR, UK; ^18^Cardiovascular and Metabolic Sciences, Max Delbrück Center for Molecular Medicine in the Helmholtz Association (MDC), Robert-Rossle Strasse 10, 13125 Berlin, Germany; ^19^DZHK (German Centre for Cardiovascular Research), Partner Site Berlin, 13347 Berlin, Germany; ^20^Charité-Universitätsmedizin, 10117 Berlin, Germany; ^21^Berlin Institute of Health (BIH), 10178 Berlin, Germany; *These authors contributed equally to this work.

^¶^Correspondence to Enrico Petretto ([enrico.petretto@duke-nus.edu.sg](mailto:enrico.petretto@duke-nus.edu.sg))

*Address for correspondence*: Duke-NUS Medical School, 8 College Road, 169857 Singapore, Republic of Singapore; Fax: (65) 62212354; Tel: (65) 66015114

**CONTENTS**

**Supplementary Figures (1-18)**

**Supplementary Tables (1-2)**

**Supplementary Notes**

**Supplementary Methods**

**Supplementary References**

**Supplementary Figures**

**Supplementary Figure 1**


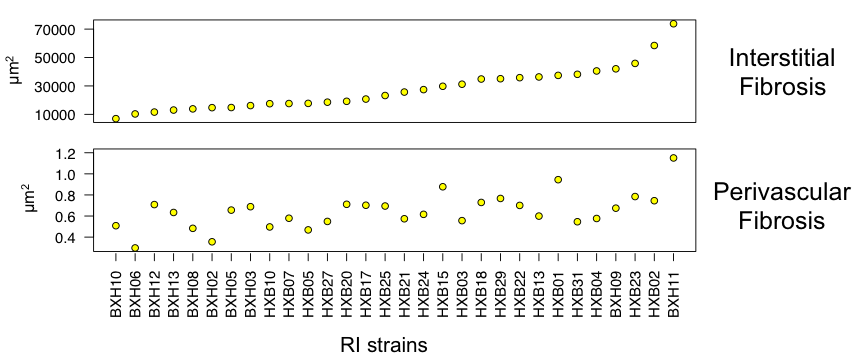


**Supplementary Figure 1. Mean interstitial and perivascular fibrosis values in each rat recombinant inbred (RI) strain.** The RI strains are sorted from low to high mean values of interstitial fibrosis. These values are also included in Supplementary Table 1c.

**Supplementary Figure 2**


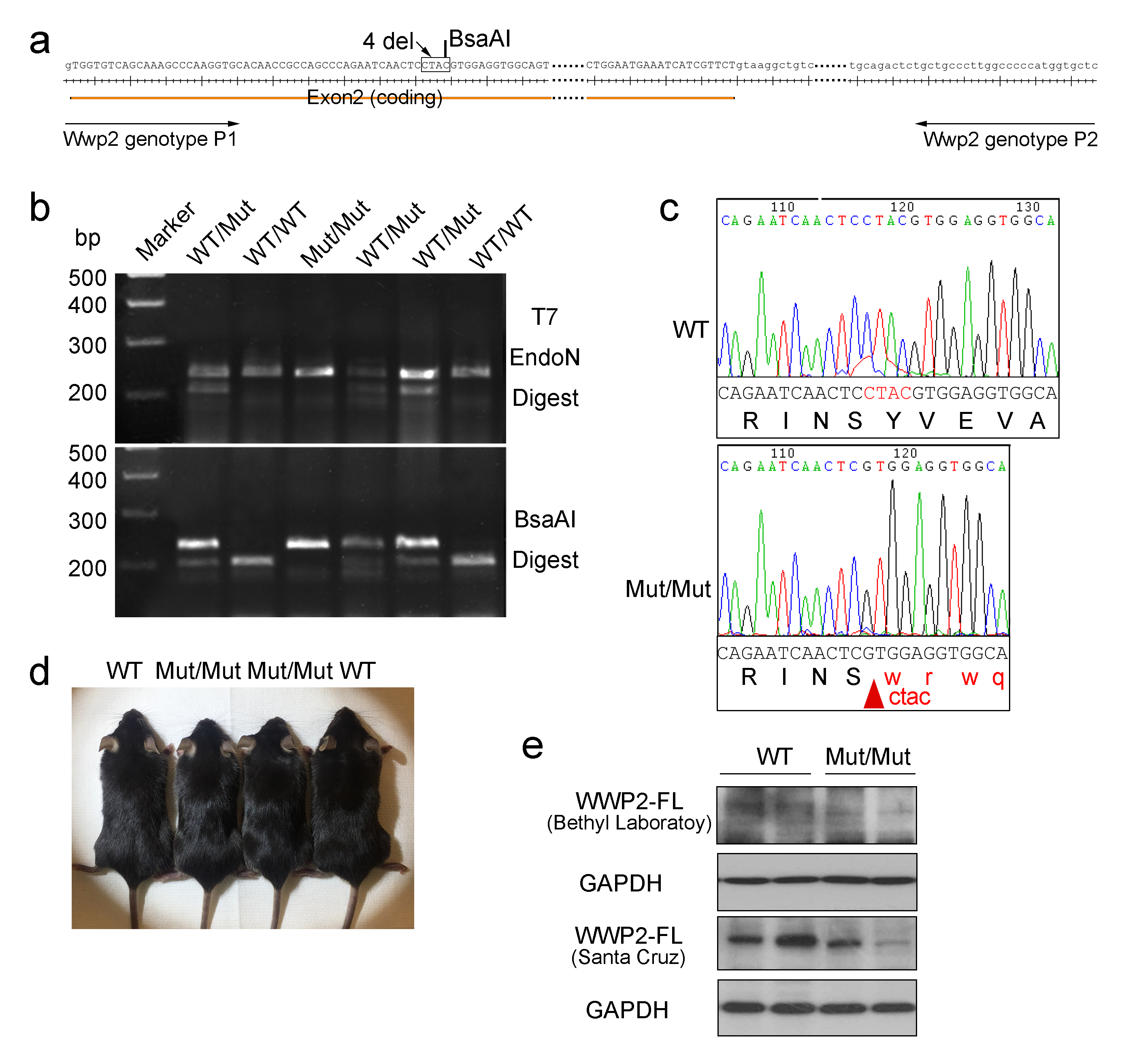


**Supplementary Figure 2. Construction of WWP2^Mut/Mut^** **mice.** (**a**) Schematic representation of the position of 4-bp deletion in the exon 2 of WWP2, with a recognition site of BsaAI. (**b**) Representative agarose gel electrophoresis image of T7 and BsaAI restriction digest. The genotype results are labeled at the top. (**c**) Sanger sequencing showing the 4bp (ctac) deletion in Mut/Mut allele. (**d**) Photograph of 9-week-old male WT and WWP2^Mut/Mut^ mice. (**e**) Western blot in cardiac (myo)fibroblasts from WT and WWP2^Mut/Mut^ mice showing a lack of the right WWP2-FL (~110 kD) band with instead detection of an aberrant short-band. This experiment was carried out using two different anti-WWP2 antibodies bought from two companies.

**Supplementary Figure 3**


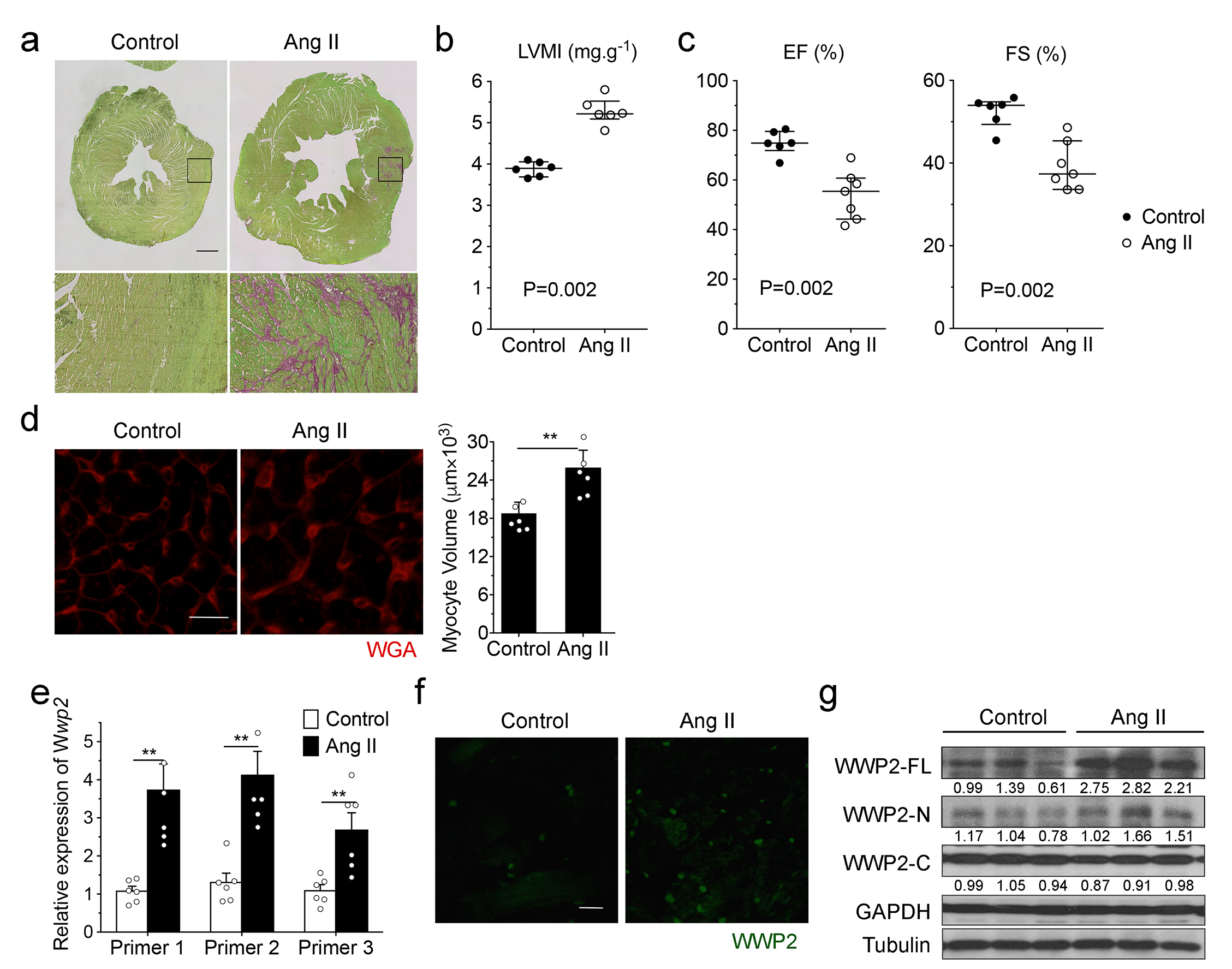


**Supplementary Figure 3. Upregulation of WWP2 and cardiac fibrosis following Ang II fusion.** (**a**) Representative Sirius Red stained sections form Ang II infused hearts revealed increased fibrosis (red) in comparison with control hearts. Scale bar: 0.5mm. (**b-c**) Increased left ventricular mass index (LVMI), and decreased ejection fraction (EF%) and fractional shortening (FS%) in mice induced by Ang II infusion for 28 days. (Mann-Whitney U test, means ± SD) (**d**) Under WGA staining (red), Ang II infused hearts show hypertrophic myocytes (left) with higher mean cell volume (right, 6 biological replicates, 8 images each, Mann-Whitney U test, means ± SD). Scale bar: 20 μm. (**e**) Control and Ang II infused mice analysis of *Wwp2* transcript level by qPCR with different sets of primers tagging different *Wwp2* isoforms (i.e. P1 tags *Wwp2*-N/FL, P2 tags *Wwp2*-FL and P3 tags *Wwp2*-C/FL). Mann-Whitney U test, n=6 for each genotype; means ± SD (**f**) Immunofluorescence showed increased WWP2 positive cells (green) in heart sections from Ang II infused mice. Scale bar: 100 μm. (**g**) Western blot analysis showing the expression of different isoforms, with increased expression WWP2-FL, WWP2-N and similar levels of WWP2-C in the Ang II infused mice, as compared with control mice.

**Supplementary Figure 4**


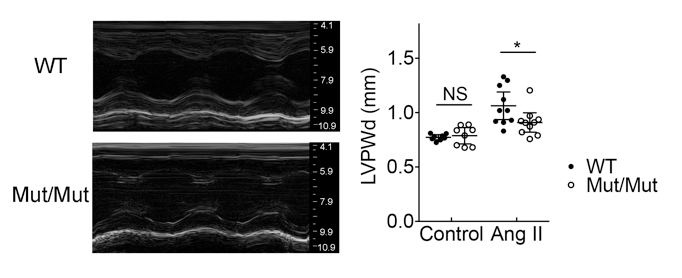
 a b

**Supplementary Figure 4. WWP2^Mut/Mut^** **mice show less Ang II-induced cardiac hypertrophy.** (**a**) Representative M-mode echocardiograms (parasternal short-axis, middle) in WT and Wwp2^mut/mut^ mice following Ang II infusion. (**b**) Cardiac echocardiogram-based analysis of left ventricular posterior wall thickness during end diastole in WWP2^mut/mut^ mice and WT mice after Ang II infusion. Left ventricular posterior wall thickness during end diastole was reduced in the WWP2^mut/mut^ mice (Mann-Whitney U test; means ± SD)

**Supplementary Figure 5**


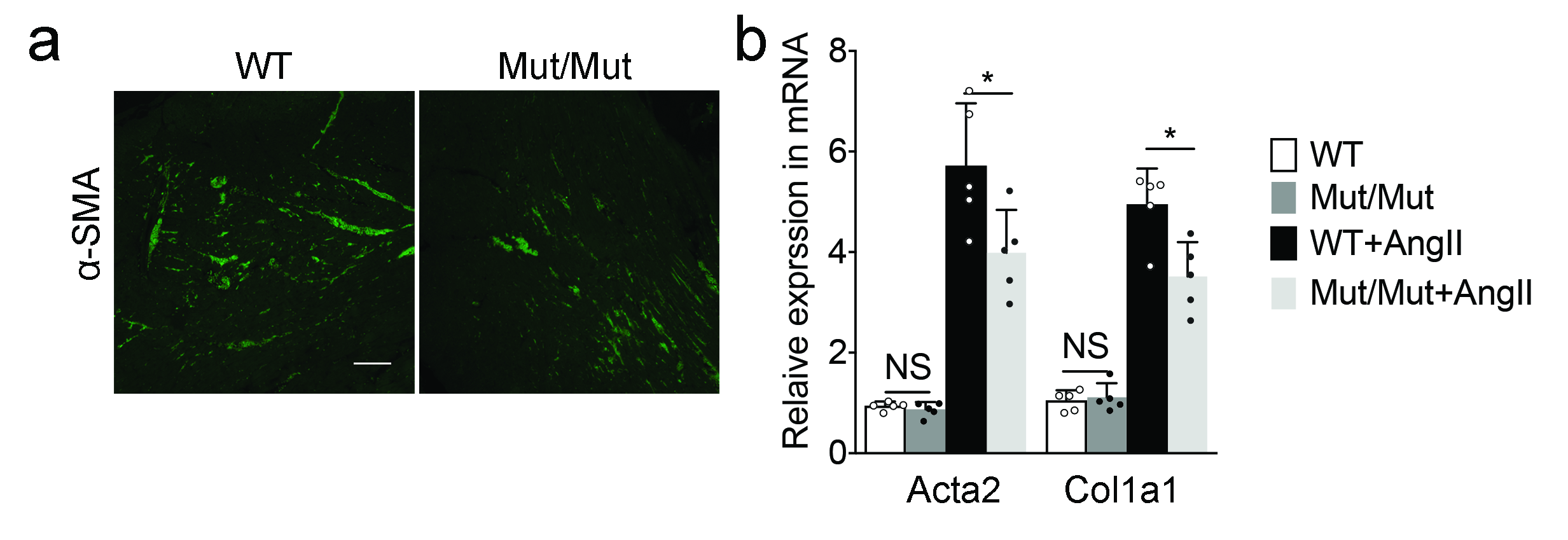


**Supplementary Figure 5. ECM features associated with WWP2^Mut/Mut^ *in vivo*. (a)** Representative immunostaining showing ACTA2 in both WT and WWP2^Mut/Mut^ mice following AngII-infusion. Scale bar: 400 µm. **(b)** Relative mRNA expression of *Acta2* and *Col1a1* in LV from WT and WWP2^Mut/Mut^ mice with Ang II infusion. mRNA expression was normalized to the level of 18S rRNA. (Mann-Whitney U test, n=5 for each group; means ± SD)

**Supplementary Figure 6**


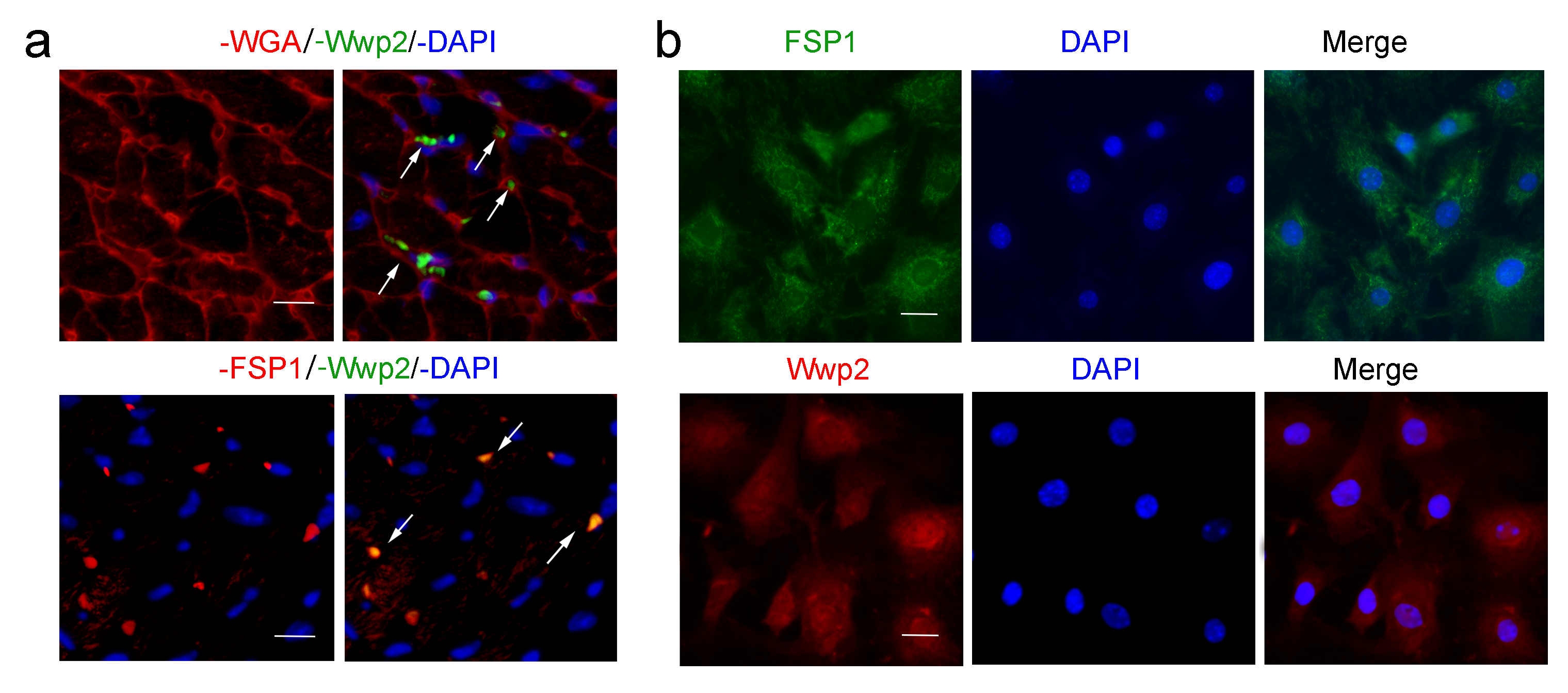


**Supplementary Figure 6. Localization and identification of WWP2 expression in heart and primary cardiac (myo)fibroblasts.** (**a**) Immunofluorescence images of LV section staining from WT LV with Ang II showed that WWP2 (green, arrow) is expressed in non-myocytes (top), and co-localized with some of the FSP1 positive cells (red, below). Note: the antibody used here is against the C-terminal region of WWP2 (Aviva Systems Biology, #ARP43089_P050). Images showing the antibody targeting the N-terminal region of WWP2 (Santa Cruz biotechnology, #sc30052) are shown in Fig. 4a. Scale bar: 40 μm. (**b**) Immunofluorescence images showing both FSP1 (green, top) and WWP2 (red, below) positive staining in primary WT cardiac (myo)fibroblasts. Scale bar: 10 μm.

**Supplementary Figure 7**


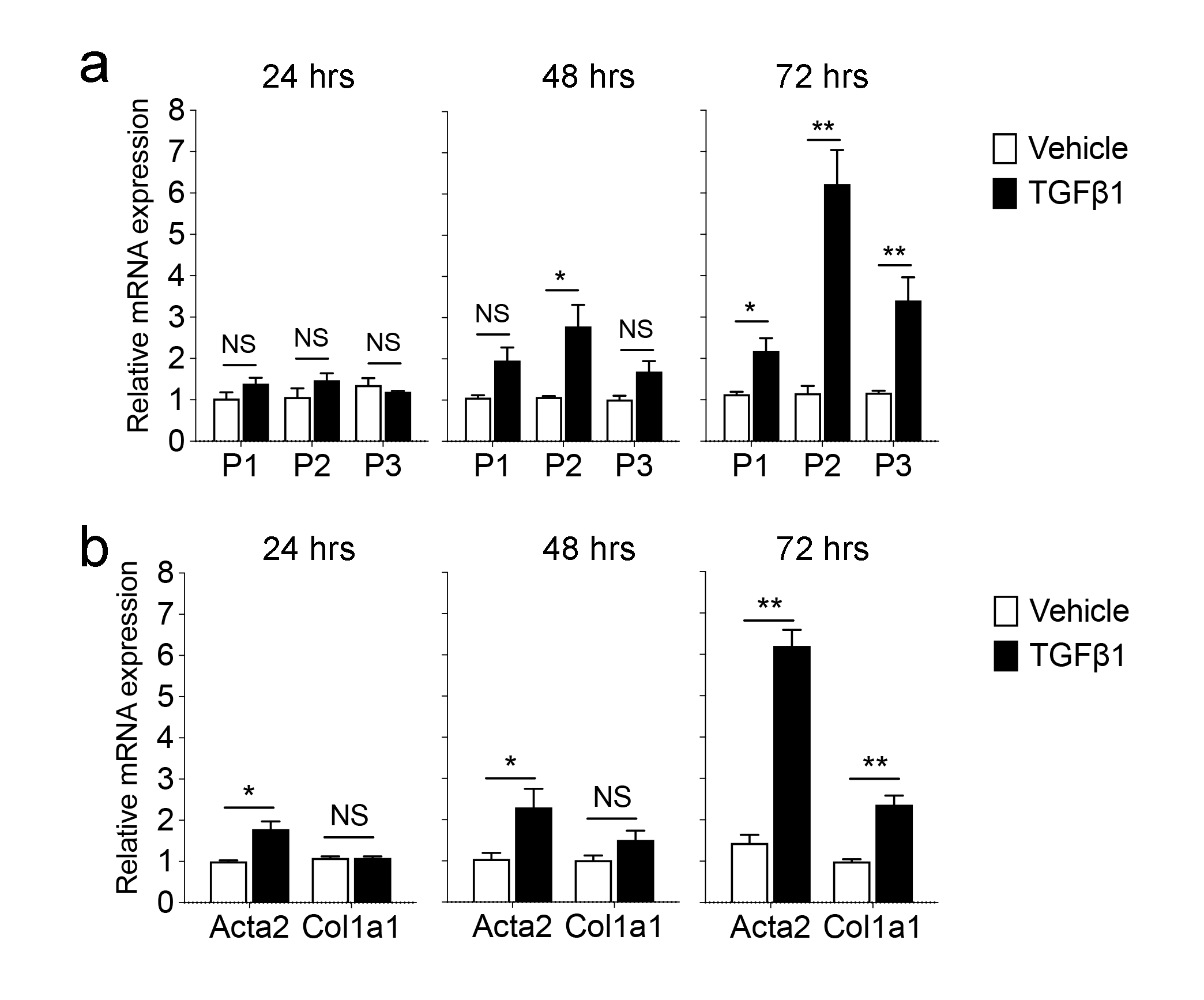


**Supplementary Figure 7. TGFβ1 response in primary cardiac fibroblasts.** (**a**) Relative mRNA expression targeting different parts of *Wwp2* isoforms (i.e. P1 tags both *Wwp2*-N and *Wwp2-*FL, P2 tags *Wwp2*-FL only and P3 tags both *Wwp2*-C and *Wwp2*-FL. The position of primers was labeled in Fig. 3a) in WT cardiac (myo)fibroblasts treated with TGFβ1 (5ng/ml) for 24, 48 and 72hrs. mRNA expression was normalized to 18S level. (Mann-Whitney U test, n=4 for each group; means ± SD) (**b**) Relative mRNA expression of *Acta1* and *Col1a1* in WT cardiac (myo)fibroblasts after TGFβ1 treatment (5ng/ml) for 24, 48 and 72hrs. mRNA expression was normalized to 18S level. (Mann-Whitney U test, n=4 for each group; means ± SD)

**Supplementary Figure 8**


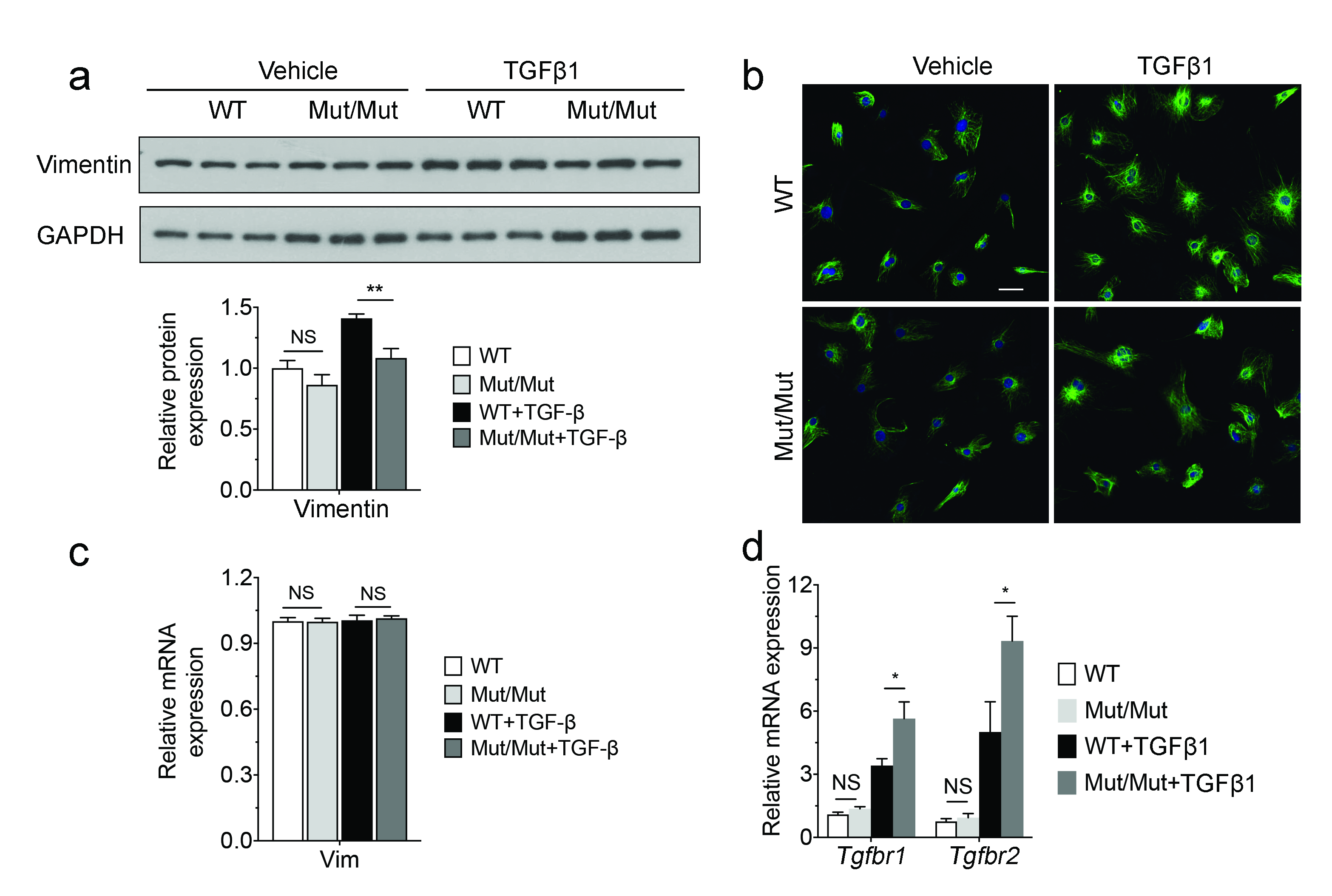


**Supplementary Figure 8. Vimentin expression level in primary cardiac fibroblasts (WT and WWP2^Mut/Mut^).** (**a**) Western blot of Vimentin in primary cardiac (myo)fibroblasts revealed that TGFβ1 treatment (5ng/ml, 72h). relatively increased the protein levels of vimentin, which was prevented in the WWP2^Mut/Mut^ cells. Representative image (top) and corresponding relative levels of Vimentin (down) (Mann-Whitney U test, n=3 for each group; means ± SD) (**b**) Representative immunostaining showing Vimentin in cardiac (myo)fibroblasts (WT and WWP2^Mut/Mut^) after TGFβ1 treatment (5ng/ml, 72h). (**c**) Relative mRNA expression of vimentin in cardiac (myo)fibroblasts (WT and WWP2^Mut/Mut^) after TGFβ1 treatment (5ng/ml, 72h). mRNA expression was normalized to 18S level. (Mann-Whitney U test, n=5 for each group; means ± SD) (**d**) Relative mRNA expression of genes encoding for TGFβ receptors in primary cardiac (myo)fibroblasts. mRNA expression was normalized to 18S level. Mann-Whitney U test, n=6 for each group; means ± SD.

**Supplementary Figure 9**

**
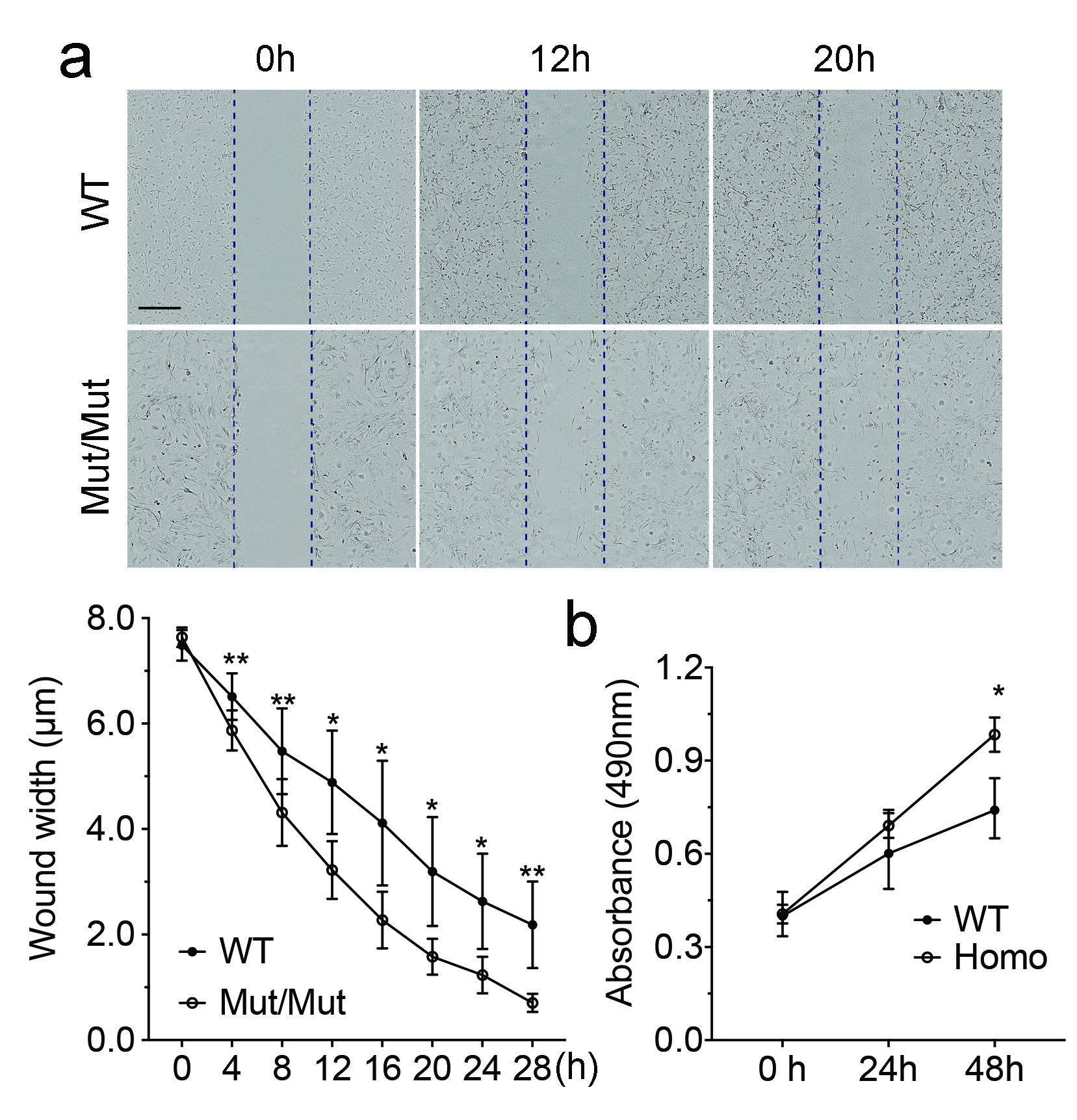
**

**Supplementary Figure 9. Proliferative and migrative feature of cardiac fibroblasts.** (**a**) Representative image (upper) and quantification (bottom) of wound closure scratch assay in a monolayer of cardiac (myo)fibroblasts from both WT and WWP2^Mut/Mut^ (myo)fibroblasts (Mann-Whitney U test, n=8 for each group; means ± SD) (**b**) MTS assay on primary cardiac (myo)fibroblasts from both WT and WWP2^Mut/Mut^ and absorbance value (490 nm) used to indicate cell number. (Mann-Whitney U test, n=8 for each group; means ± SD).

**Supplementary Figure 10**

**
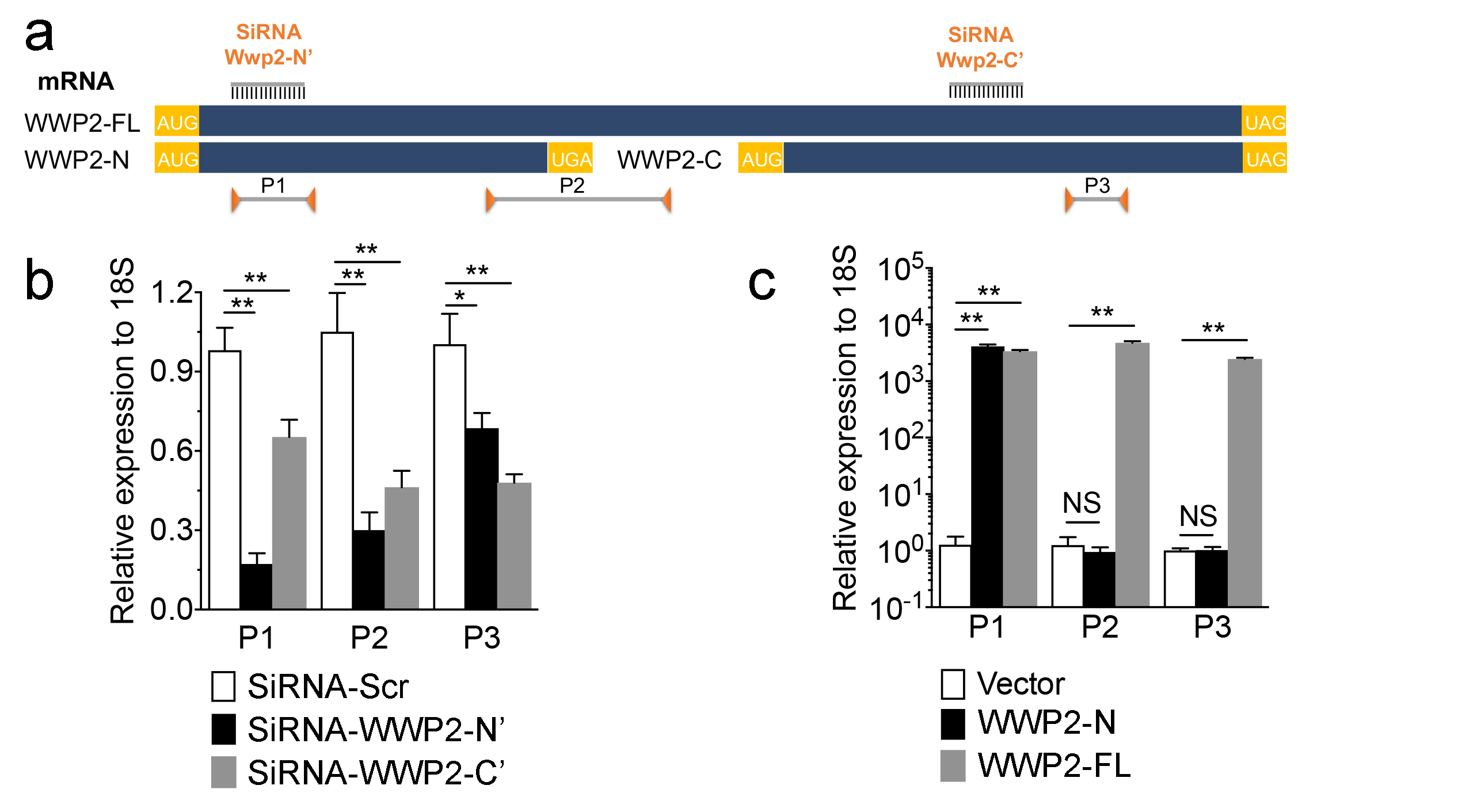
**

**Supplementary Figure 10.** (**a**) Schematic representation of SiRNAs (*siRNA-Wwp2-N'* and *siRNA-Wwp2-C'*) targeting different parts of *Wwp2* mRNA. Position of the three primer pairs (P1, P2 and P3, tagging *Wwp2*-N/FL, *Wwp2*-FL and *Wwp2*-C/FL, respectively), used for the quantification of *Wwp2* expression levels are also labeled. (**b**) SiRNA targeting *Wwp2* (**c**) and Vector containing *Wwp2-N* and *Wwp2-FL* isoforms in murine cardiac (myo)fibroblasts after 72 hrs of TGFβ1 stimulation. (Mann-Whitney U test, n=6 for each genotype; means ± SD).

**Supplementary Figure 11**


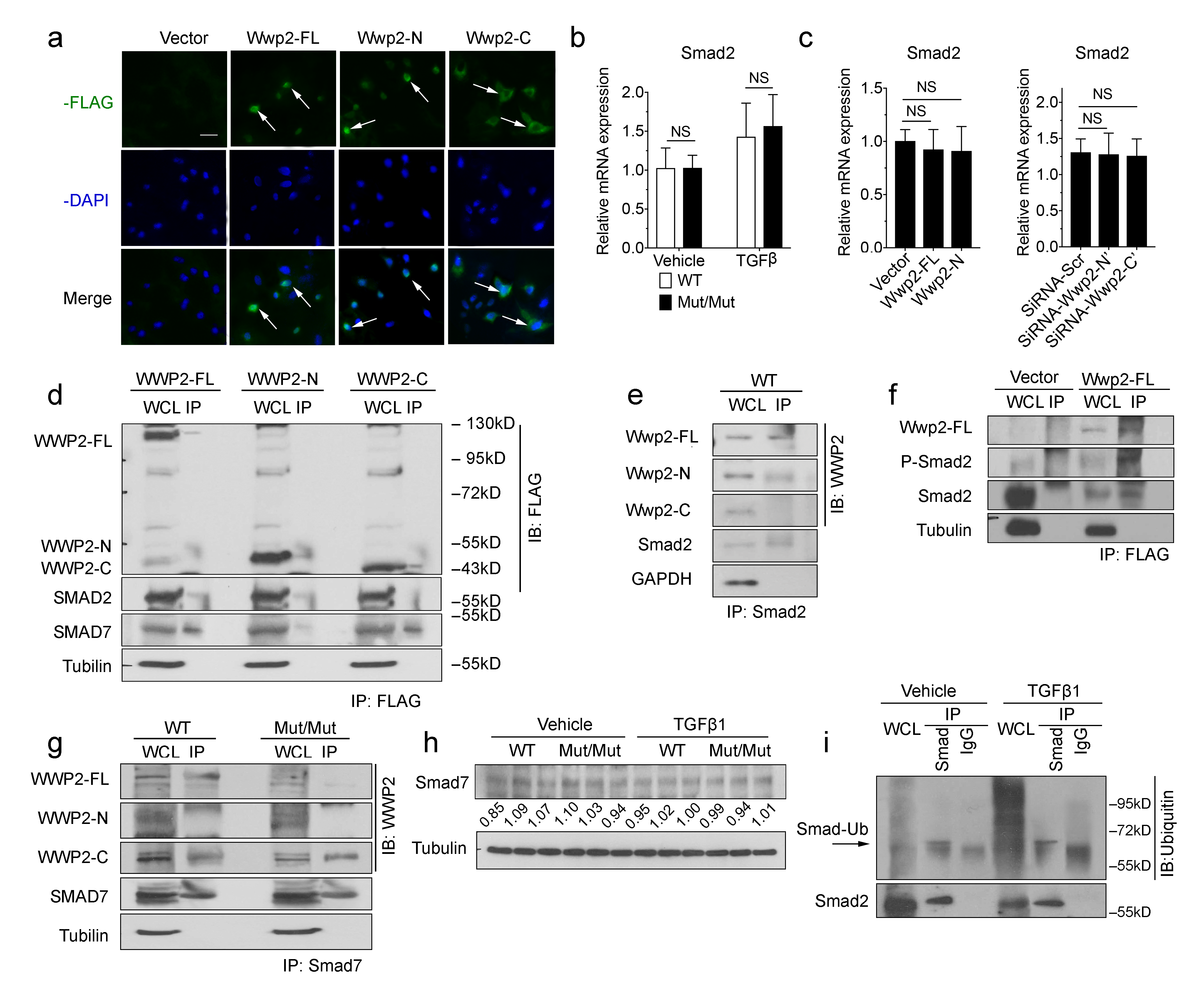


**Supplementary Figure 11. Selective interaction of SMAD2 with individual WWP2 isoforms.** (**a**) Representative immunofluorescences showing the distribution of individual WWP2 isoforms. WWP2 isoforms with FLAG tag were introduced into NIH-3T3 fibroblast cells, and immunofluorescence against the FLAG antibodies were performed. scale bar, 40 μm. (**b-c**) *Smad2* expression in fibroblast cells under TGFβ1 stimulation (**b**), SiRNA targeting *Wwp2* (c, left) and Vector containing *Wwp2* (c, right) in murine cardiac fibroblasts. (Mann-Whitney U test, n=6 for each genotype; means ± SD). (**d**) NIH-3T3 cells were transfected with WWP2-Flag isoforms and co-immunoprecipitation experiment shows a direct interaction of SMAD2 or SMAD7 with WWP2 isoforms. Lysates were subject to immunoprecipitation with anti-FLAG antibodies, followed by western blotting probed with antibodies as indicated. (**e**) Co-immunoprecipitation experiment shows a direct interaction of SMAD2 with WWP2-FL and -N isoforms in WT (myo)fibroblasts following TGFβ1 (5ng/ml, 16hr). Lysates prepared from (myo)fibroblasts were subject to immunoprecipitation with anti-SMAD2 antibodies, followed by western blotting probed with antibodies as indicated. (**f**) Co-immunoprecipitation experiment shows a direct interaction of p-SMAD2 with WWP2-FL in NIH-3T3 cells transfection with WWP2-FL after TGFβ1 treatment (5ng/ml, 16hr). (**g**) Co-immunoprecipitation experiment shows a direct interaction between SMAD7 and WWP2-C isoforms in both WT and WWP2^Mut/Mut^ (myo)fibroblasts. Lysates prepared from (myo)fibroblasts from both WT and WWP2^Mut/Mut^ were subject to immunoprecipitation with anti-SMAD7 antibodies, followed by western blotting probed with antibodies as indicated. (**h**) Representative Western blot analysis of SMAD7 protein levels in WT and WWP2^Mut/Mut^ (myo)fibroblasts with/without TGFβ1. (**i**) In-cell ubiquitylation of SMAD2 by immunoprecipitation in NIH-3T3 fibroblasts. Cells were treated with TGFβ1 (5ng/ml, 16 hr) and then MG132 (10 μM, 4hr), then lysates were prepared and subject to immunoprecipitation with anti-SMAD antibodies, followed by western blotting probed with antibodies as indicated.

**Supplementary Figure 12**


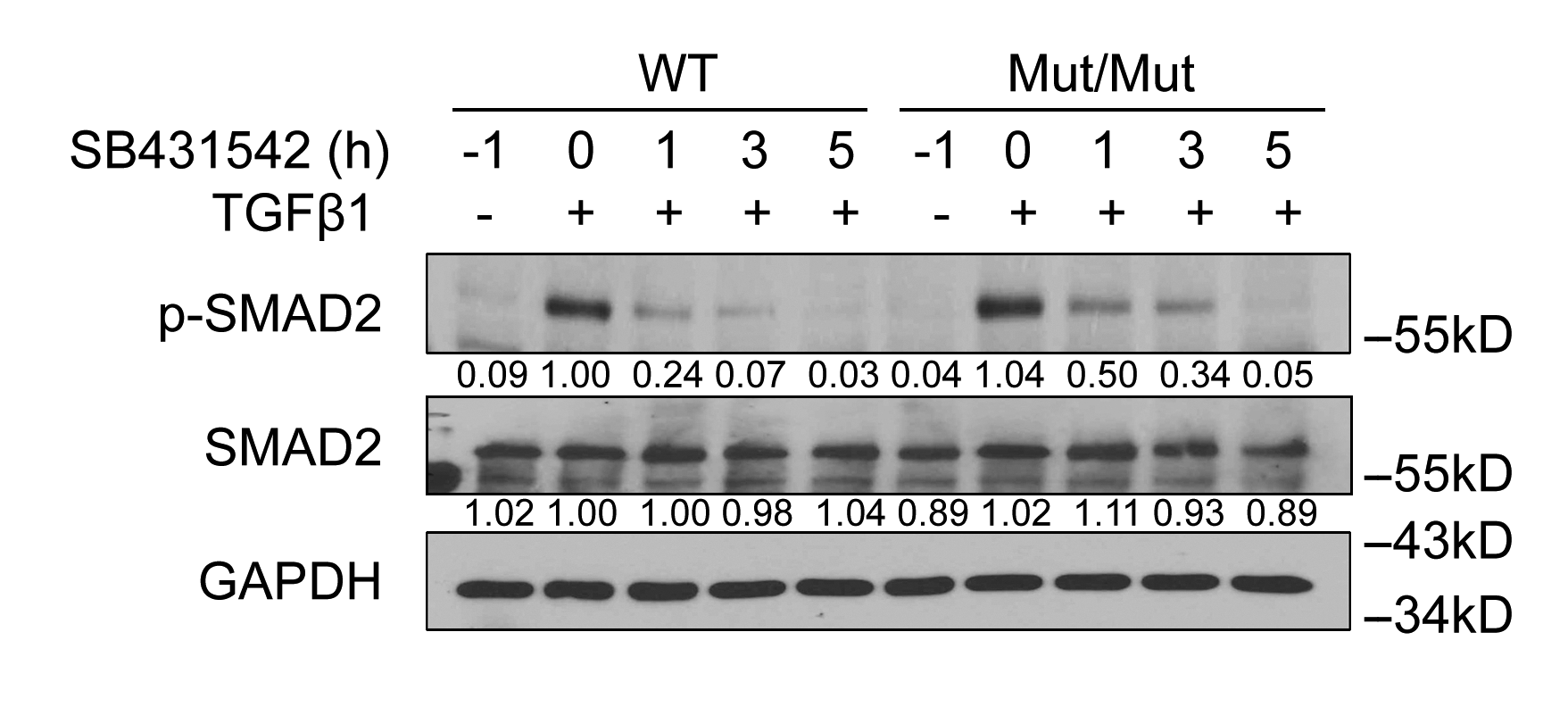


**Supplementary Figure 12.** Western Blotting of p-SMAD2 and SMAD in WT and WWP2^mut/mut^ (myo)fibroblasts treated with TGFβ1 for 1hr and then with SB431542 for 5hr.

**Supplementary Figure 13**

**
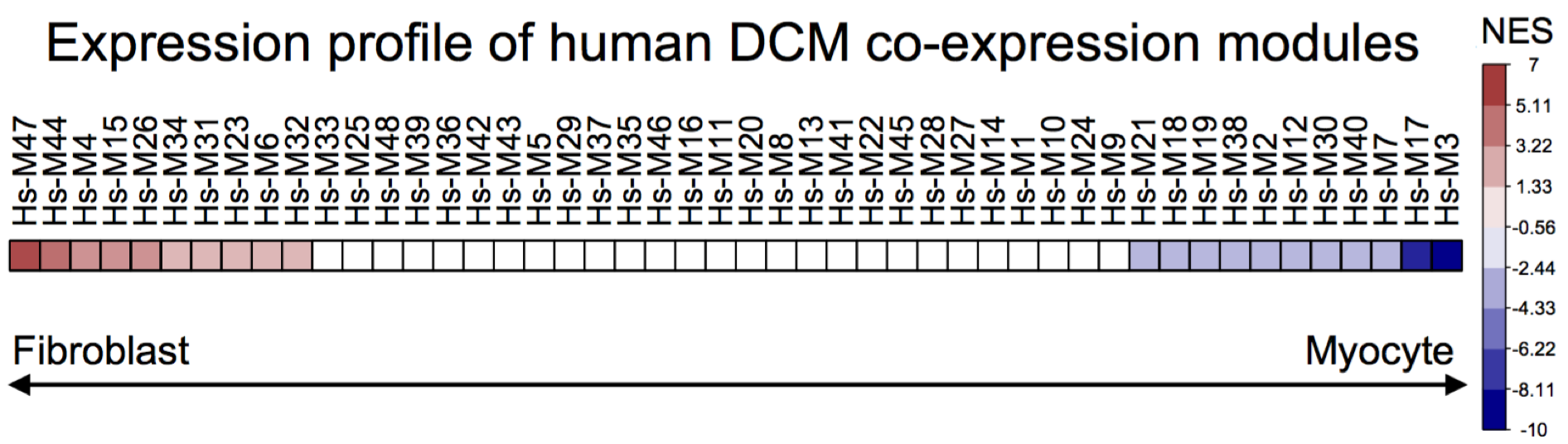
**

**Supplementary Figure 13.** **Cardiac fibroblast-to-myocyte fold change GSEA test in human DCM co-expression networks.** We ran a test to assess whether any of the human DCM networks were overrepresented for genes with higher or lower expression levels in cardiac fibroblasts when comparing to cardiomyocytes. More specifically, we carried out a GSEA analysis of cell type gene expression data from primary mouse cardiac fibroblasts and myocytes. After computing a fibroblast-to-myocyte expression fold change, we ran GSEA ranking all the human genes (mapped to their corresponding mouse ortholog) by their cardiac fibroblast-to-myocyte expression fold change. Then we used the human DCM clusters as gene sets to test for overrepresentation of genes present at the top or bottom of this ranked list. The level plot displays the NES (NES represents the strength of enrichment normalized by gene set size) of GSEA enrichment for fold change of cardiac fibroblast-to-myocyte expression in each of the human DCM co-expression networks inferred in LV tissue. All the human DCM networks inferred in left ventricle are presented sorted by the NES value (from left to right, highest to lowest). In this analysis, high values of NES imply enrichment for genes that are more expressed in cardiac fibroblasts than in myocytes, whereas low values of NES represent overrepresentation of genes in the cluster that are more highly expressed in myocytes than in cardiac fibroblasts. White denotes non-significant enrichment in the cluster (adjusted p-value > 0.05), significant enrichments are depicted in non-white. Red denotes positive values of NES, while blue denotes negative values of NES. With this analysis we can inspect whether genes present in the human DCM clusters are enriched for genes that are more highly expressed in cardiac fibroblast than myocytes or the opposite. This analysis can potentially uncover fibroblasts- or myocytes-specific co-expression networks. Out of the 48 human co-expression networks, the Hs-M47 (hECM-network) was the most enriched network for genes with fibroblast expression.

**Supplementary Figure 14**

**
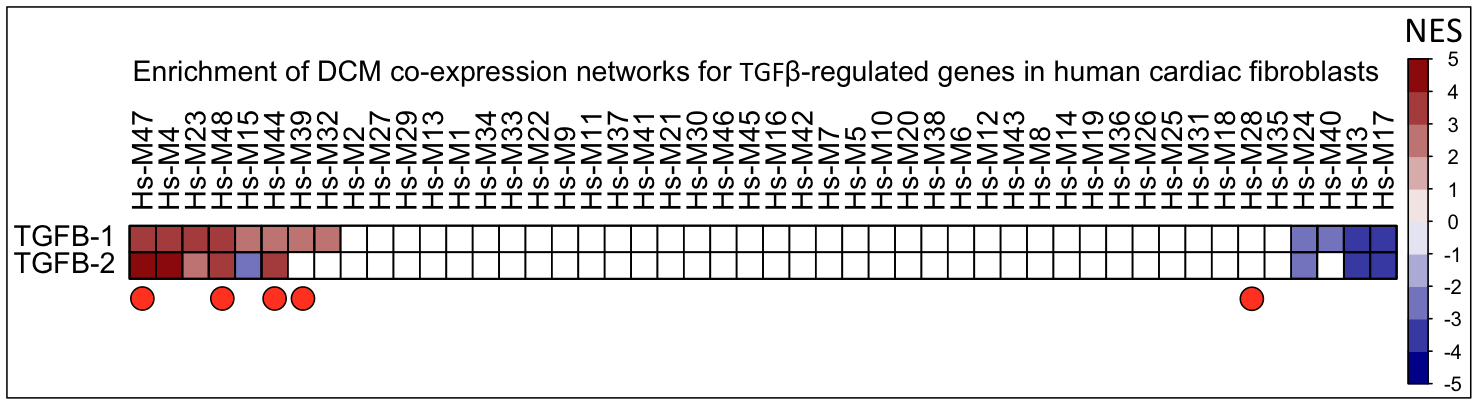
**

**Supplementary Figure 14. Enrichment of DE genes (24h TGFβ induction) in the human DCM co-expression networks.** Human cardiac (myo)fibroblasts were incubated for 24hrs with either TGFβ1 (n=3) or TGFβ2 (n=3). DE genes were computed by comparing the RNA-seq counts of the TGFβ1- and TGFβ2-stimulated (myo)fibroblasts with control (myo)fibroblasts at 24hrs. The DESeq2 differential expression test statistic was used as ranking measure for running GSEA by using the human DCM LV co-expression networks as gene sets to test for enrichment. Level plot displaying the results of the GSEA analysis of all the human DCM LV co-expression networks testing for overrepresentation within the network genes of genes up- and down- regulated by TGFβ1 and/or TGFβ2 in human cardiac (myo)fibroblasts. DCM networks are displayed sorted by TGFβ1 NES value (NES or strength of enrichment normalised by gene set size) from high (left) to low (right) NES. Non-significant results (adjusted p-value > 0.05) are shown in white. Positive significant NES values are shown in red, whereas negative significant NES values are shown in blue. Red dots highlight the five prioritised human fibrotic LV co-expression networks.

**Supplementary Figure 15**

**Supplementary Figure 15 Conservation of prioritised human cardiac co-expression networks in right ventricle tissue.** Adjusted empirical p-values (adjusted for the number of networks tested, n=48) are shown for the five prioritised human DCM LV co-expression networks.

**Supplementary Figure 16**

**Supplementary Figure 16.** **Genome-wide mapping of prioritised rat LV co-expression networks in the RI strains.** The mapping of the selected rat networks was carried out using HESS (multivariate Bayesian regression approach) and a panel of 1,384 SNPs. For each rat chromosome (1-20, x-axes), the strength of evidence for a SNP being a regulatory *hotspot* for the rat network expression in heart is measured by the median (interquartile range also shown, IQR) of the Bayes Factor (y-axes). (**a**) Rat gene network M1 (n=219 genes). HESS identified a locus on rat chromosome 19q12 (spanning 6.22 Mb). (**b**) Rat network M2 (n=645 genes). This network mapped to a locus in rat chromosome 4q13 (spanning 4.39Mb). (**c**) Rat network M12 (n=313 genes). This network did not present any strong evidence (median BF cluster > 100) to be associated with any regulatory locus in the rat genome.

**Supplementary Figure 17**

**Supplementary Figure 17. Overview of the loci associated with the regulatory loci identified in the rat heart.** From Rat Ensembl 69 browser view. (**a**) Regulatory locus in rat chromosome 19q12 (spanning 6.22 Mb) associated with the gene expression levels of the rat co-expression network M1 (n=219 genes). (**b**) Regulatory locus in rat chromosome 4q12 (spanning 4.39Mb) associated with the gene expression levels of the rat co-expression network M2 (n=645 genes).

**Supplementary Figure 18：Original blot of Figure 3**

**Supplementary Figure 18：Original blot of Figure 3**

**Supplementary Figure 18：Original blot of Figure 4**

**Supplementary Figure 18：Original blot of Figure 4**

**Supplementary Figure 18：Original blot of Figure 4**

**Supplementary Figure 18：Original blot of Figure 5**

**Supplementary Figure 18：Original blot of Figure 5**

**Supplementary Figure 18：Original blot of Figure 5**

**Supplementary Figure 18：Original blot of Figure 5**

**Supplementary Figure 18：Original blot of Supplementary Figure 2**

**Supplementary Figure 18：Original blot of Supplementary Figure 3**

**Supplementary Figure 18：Original blot of Supplementary Figure 8**

**Supplementary Figure 18：Original blot of Supplementary Figure 11**

**Supplementary Figure 18：Original blot of Supplementary Figure 11**

**Supplementary Figure 18：Original blot of Supplementary Figure 11**

**Supplementary Figure 18：Original blot of Supplementary Figure 11**

**Supplementary Figure 18：Original blot of Supplementary Figure 11**

**Supplementary Figure 18：Original blot of Supplementary Figure 11**

**Supplementary Figure 18：Original blot of Supplementary Figure 11**

**Supplementary Figure 18：Original blot of Supplementary Figure 11**

**Supplementary Figure 18：Original blot of Supplementary Figure 12**

**Supplementary Tables**

**Supplementary Table 1. Clinical information available of the 27 rTOF patients from whom RV tissue was collected.**

| **TOF samples (n=27)** |  |  |
| --- | --- | --- |
| Age, years | 32.0 ± 10.6 |  |
| Gender (Male/Female) | 21/6 |  |
| rTOF with associated lesions * | 2 |  |
| Presence of symptoms, n(%) | 17 (63) |  |
| Predominant haemodynamic lesion - PR/PS/Both | 25/1/1 |  |
| Heart rate, bpm | 72 ± 10 |  |
| Systolic blood pressure, mmHg | 117 ± 11 |  |
| Diastolic blood pressure, mmHg | 71 ± 9 |  |
| QRS duration on ECG, ms | 152 ± 23 |  |
| Cardiothoracic Ratio | 0.54 ± 0.05 |  |
| **Previous palliative procedures, n(%)** | 12 patients with 14 procedures |  |
| Blalock-Taussig shunt | 8 |  |
| Waterston shunt | 4 |  |
| Brock procedure | 1 |  |
| Open pulmonary valvotomy | 1 |  |
| **Characteristics of initial repair** |  |  |
| Age, years | 4.2 ± 3.4 |  |
| Transannular patch (%) | 14 (52) |  |
| RVOT patch (%) | 9 (33) |  |
| RV to PA conduit (%) | 3 (11) |  |
| Surgical details not known (%) | 1 (4) |  |
| Restrictive RV physiology at echo, n (%) | 6 (22) |  |
| **CMR at study recruitment** |  |  |
| RVEDVi, ml/m^2^ | 146.8 ± 36.6 |  |
| RVESVi, ml/m^2^ | 73.2 ± 26.3 |  |
| RVSVi, ml/m^2^ | 73.5 ± 20.3 |  |
| RVEF, % | 50.9 ± 9.8 |  |
| cRVEF, % | 28.7 ± 9.1 |  |
| RVMi, g/m^2.^ | 62.6 ± 14.2 |  |
| Pulmonary regurgitant fraction, % | 38.2 ± 12.4 |  |
| RAAi, cm^2^/m^2^ | 14.8 ± 3.3 |  |
| LVEDVi, ml/m^2^ | 78.9 ± 17.7 |  |
| LVESVi, ml/m^2^ | 31.6 ± 12.7 |  |
| LVSVi, ml/m^2^ | 47.2 ± 8.7 |  |
| LVEF, % | 60.7 ± 7.6 |  |
| LVMi, g/m^2^ | 65.5 ±14.4 |  |
| LAAi, cm^2^/m^2^ | 9.4 ± 2.1 |  |
| RV LGE score ^†^ | 4.9 ± 2.1 |  |
| * 1 pulmonary atresia, 1 absent pulmonary valve. ^†^ RV/LV insertion points not included.  ± standard deviation. | | |

**Supplementary Table 2**. Primers for quantitative reverse transcription PCR detection.

| Host | Gene | Forward (5’-3’) | Reverse (3’-5’) |
| --- | --- | --- | --- |
| Mouse | *Wwp2*-P1 | TTTGAGAAGTCCCAGCTTACCC | CTCCAGACCTTCAGATCCAAATG |
|  | *Wwp2*-P2 | ACCAAGAGCAGCAAGCAGAT | GTCTCCTCGATGGCGTACAG |
|  | *18S rRNA* | GTAACCCGTTGAACCCCATT | CCATCCAATCGGTAGTAGCG |
|  | *Tgfbr1* | CTCCCGTGGCTTCTAGTGC | GCCTTAGTTTGGACAGGATCTG |
|  | *Tgfbr2* | CATTTGGTTCCAAGGTGC | TGGTAGTGTTCAGCGAG |
|  | *Acta2* | GTCCCAGACATCAGGGAGTAA | TCGGATACTTCAGCGTCAGGA |
|  | *Smad2* | AATACGGTAGATCAGTGGGACA | CAGTTTTCGATTGCCTTGAGC |
|  | *Col1a1* | GCTCCTCTTAGGGGCCACT | CCACGTCTCACCATTGGGG |
|  | *EDA-FN* | CCCTAAAGGACTGGCATTCA | TCTGCAGTGTCGTCTTCACC |
|  | *TCF21* | CCCACTAAGAAAAGCCCGCTC | CCGTTCTCGTACTTGTCGTTG |
|  | *Postn* | CCTGCCCTTATATGCTCTGCT | AAACATGGTCAATAGGCATCACT |
|  | *CTGF* | CCTGCCCTTATATGCTCTGCT | AAACATGGTCAATAGGCATCACT |
| Human | *WWP2*-P1 | GAGAAACTGTCCGTTCGGGTG | GACAAGCGCGTCCTCAATGAT |
|  | *WWP2*-P2 | CTTGTCGTGTCGTTCCGCTT | ACCATTTGCTTCACACTTGACTT |
|  | *COL1A1* | GTGCGATGACGTGATCTGTGA | CGGTGGTTTCTTGGTCGGT |
|  | *COL1A2* | GAGCGGTAACAAGGGTGAGC | CTTCCCCATTAGGGCCTCTC |
|  | *LUM* | CTGCGTTTATCTCACAACGAACT | CAGATCCAGCTCAACCAGGG |
|  | 18S rRNA | GGCCCTGTAATTGGAATGAGTC | CCAAGATCCAACTACGAGCTT |

The primers used for genotyping:

|  | Sequence |
| --- | --- |
| Forward (5’-3’) | GTGGTGTCAGCAAAGCCCAAGGTGC |
| Reverse (5’-3’) | GAGCACCATGGGGGCCAAGGGCAGC |

**Supplementary Notes**

**﻿ Supplementary Note 1***.* **Prioritization of cross species fibrotic co-expression networks**

Comparative genomics can be used to highlight relevant functional processes conserved across species and thus to aid dissecting the genetics of complex disease. With the aim of prioritising co-expression networks associated with fibrotic processes relevant in myocardial disease for further and more in-depth analyses, we intersected the rat co-expression networks with the human co-expression networks built in the DCM patients. The human networks that were significantly intersecting to any of the rat networks can be seen in Fig. 1.

The human network that presented the highest degree of intersection with a rat network was Hs-M17 (intersecting to the rat network M31 adjusted p-value= 4.5 × 10^-77^). Hs-M17 shared 68 genes (11.4% of the human cluster) with the rat network M31 (these 68 genes represent a 67% of the rat cluster). These clusters were both enriched for immune processes-related genes (Supplementary Table 1 and 2).

In addition, four human clusters presented a high degree of intersection (adjusted p-value < 10^-30^) with a rat cluster:

- **Hs-M40**: intersecting to the rat network M13 with adjusted p-value= 2.2 × 10^-46^. Hs-M40 and M13 were enriched for functional terms involved in immune processes.
- **Hs-M47**: intersecting to the rat network M1 with adjusted p-value= 8.4 × 10^-45^. Hs-M47 and M1 were enriched for functional terms involved in extracellular matrix.
- **Hs-M15**: intersecting to the rat network M27 with adjusted p-value= 6.8 × 10^-30^. Hs-M15 and M27 were enriched for functional terms involved in cell cycle.

Besides, we aimed to find human co-expression networks conserved in the rat heart that were associated with varying levels of fibrosis, and that can also be relevant for human myocardial disease. Therefore, we selected those DCM networks that met these two requirements:

1. **Significant intersection (after multiple correction adjustments) with any rat fibrosis-associated (perivascular, interstitial or both) co-expression network.** Out of the 48 DCM human networks, 14 networks (~29% of the total number of DCM networks) were significantly intersecting to any rat network (Fig. 1). Among these 14 human DCM networks, 9 were intersecting to any of the rat networks that were also associated with fibrosis (these 9 networks represent a ~19% of total number of DCM networks). Hence, 9 human networks passed this first selection criteria: Hs-M3, Hs-M8, Hs-M23, Hs-M28, Hs-M39, Hs-M40, Hs-M44, Hs-M47 and Hs-M48.
2. **Differential co-expression between DCM and control LV samples.** Out of the nine networks that met the previous point, five DCM networks (Hs-M28, Hs-M39, Hs-M44, Hs-M47 and Hs-M48) were also differentially co-expressed between DCM patients and control samples.

With this selection procedure, we narrowed down the 48 DCM human networks to five for further analyses. Unexpectedly, these five human networks that met all the aforementioned criteria (Hs-M28, Hs-M39, Hs-M44, Hs-M47 and Hs-M48), turned out to be intersecting to only three fibrosis-associated rat networks (M1, M2 and M12). As it can be seen in Supplementary Table 1, these three rat networks were enriched for the KEGG pathways: *“ECM-receptor interaction”* (M1), *“Insulin signalling pathway”* (M2) and *“Adherence junctions”* (M12).

Furthermore, among the five prioritised human networks, Hs-M47 (or hECM-network) was the network that shown the strongest degree of intersection with a rat network (Fig. 1). Hs-M47 had 72 genes in common (when considering rat-human one-to-one orthologs) with the rat network M1 (adjusted p-value= 8.4 × 10^-45^). These 72 genes represented a 18 % of the Hs-M47 (399 genes with one-to-one rat-human ortholog) and a 37.1 % of the rat network M1 (194 rat genes with one-to-one rat-human ortholog).

*﻿* **Supplementary Note 2. Analysis of prioritized human co-expression networks**

1. Enrichment of TGFβ responsive genes

As there is a well-established role for TGFβ in the induction of the fibrogenic response, it is of interest to explore whether any of the prioritised networks is overrepresented for genes that are transcribed in response to TGFβ induction. With this aim, we carried out a differential expression analysis with RNA-seq data from primary human cardiac (myo)fibroblasts in which we compared control samples at 24h (n=3) against two sets of primary human cardiac (myo)fibroblasts induced for 24h with either TGFβ1 (n=3) or TGFβ2 (n=3). Then, to test for overrepresentation of TGFβ differentially expressed genes within the DCM co-expression networks*,* we ran a GSEA test by querying as gene sets all the human DCM networks and supplying as ranked list, the differential expression test statistic, which represents strength of enrichment while keeping directionality.

Four of the five human DCM prioritised networks were found to be enriched for TGFβ*-*upregulated genes (Supplementary Figure 14). By contrast, the prioritised network Hs-M28 was not significantly enriched for TGFβ differentially expressed (DE) genes (adjusted p-value >0.05).

The network that presented the strongest enrichment for TGFβ*-*DE genes was Hs-M47 (TGFβ1 NES = 3.98, adjusted p-value ≤ 1×10^-4^; TGFβ2 NES = 4.07, adjusted p-value ≤ 1×10^-4^). 46% and 49% of the genes in Hs-M47 were upregulated in TGFβ1*-* or TGFβ2- stimulated (myo)fibroblasts, respectively. Among the four prioritised human networks that were enriched for TGFβ-upregulated genes, three of them (Hs-M47, Hs-M48 and Hs-M44) turned out to be enriched for genes up-regulated by both TGFβ1 and TGFβ2. Hs-M39, although it was enriched for TGFβ1-up-regulated genes, was the only network that did not show enrichment for TGFβ2- up-regulated genes.

2. Conservation of prioritised human networks in right ventricle fibrotic disease

The gene regulatory networks inferred in DCM LV could be capturing transcriptional responses that also act in right ventricle (RV) fibrotic disease. To assess this, we tested the conservation of the networks in additional transcriptomic data sets of RV.

We analysed a human cohort of RV fibrosis (n=38). This data set consists of RNA-seq expression in RV from 27 repaired Tetralogy of Fallot (rTOF) patients and 11 control samples (see Supplementary Methods).

To explore the conservation of the co-expression networks built in human DCM LV in rTOF RV, we implemented an empirical permutation test that assesses whether it is likely to find by chance each of the DCM networks in an independent data set (how likely is it to find by chance a set of genes of the same size as the network with the same value of average absolute correlation in the data set under testing). In this test: 1) a random set of genes of the same size of each of the human LV DCM networks is sampled, 2) the mean correlation of both the network being tested and the randomly sampled network is computed, 3) after repeating steps 1 and 2 for at least 10,000 times, an empirical p-value is computed for each network and adjusted for the number of networks present in LV (n=48). Full details of this procedure can be found in Supplementary Methods. For each of the 48 DCM co-expression networks, we carried out two runs of the empirical permutation test, rTOF and control separately. In this test, significant results imply that the network is conserved in RV and either rTOF or control specifically. The results of this test are presented in Supplementary Figure 15.

*﻿﻿* **Supplementary Note 3. Mapping master genetic regulators of co-expression networks**

In previous sections, unbiased co-expression networks were independently built in the rat (RI strains) and human DCM patient hearts. A subset of these rat and human networks (three rat networks: M1, M2 and M12, and five human networks: Hs-M28, Hs-M39, Hs-M44, Hs-M47 and Hs-M48) were further prioritised by following several selection criteria (i.e. conservation in rat and human, association to quantitative levels of fibrosis in the rat and conservation in human DCM heart with a pattern of co-expression not present in control samples). Here, we use multivariate Bayesian genetic mapping approaches to map these prioritised gene co-expression networks to the rat and human genomes.

In keeping with previous studies in the rat, where the BXH/HXB rat panel yielded increased power to map master genetic regulators for trans-eQTL networks ^1^, first we considered the expression of the fibrosis rat networks genes as a multivariate quantitative trait and we jointly mapped the expression levels of the genes in each of the rat networks to the rat genome. Then, we inspected whether the regulatory loci identified in the rat were independently replicated in human DCM heart by joint mapping of the genes in the human ortholog networks, to the human loci that are syntenic to the rat regulatory loci.

We started by performing genome-wide genetic regulators mapping in the RI strains of the three prioritised rat networks (M1 -*“ECM”,* M2 - *“Insulin signalling pathway”* and M12 -*“Adherence junctions”*), Supplementary Figure 16. As suggested in ^2^, only those genetic variants that yielded a median network Bayes Factor (BF) >100 were deemed as significant. M1 mapped to a single locus in rat chromosome 19 (median BF of the genes in the network = 181.66, Supplementary Figure 17a), whereas M2 mapped one locus in chromosome 4 (median BF of the genes in the network = 419.43, Supplementary Figure 17b). Nevertheless, none of the SNPs in the rat genome had enough joint evidence of association with the genes in M12 (the median BF of the genes in the M12 is < 100), Supplementary Figure 17c.

The loci associated with the regulatory loci identified for M1 and M2 are depicted in Supplementary Figure 17a and b, respectively. Within the locus in rat chromosome 19 (associated with M1) there are several rat QTLs (e.g. *“non-insulin dependent diabetes mellitus*”, “*collagen-induced arthritis*” and “*body weight*”) and ~100 protein coding genes, Supplementary Figure 17a. The locus associated with M2 was also associated with several rat QTLs and was encoding for ~30 protein coding genes, Supplementary Figure 17b. This evidences a well-known problem of genetical mapping in RI strains: although there is increased statistical power, due to the large size of the haplotype blocks (usually around 5-10Mb), the identification of the causal *master genetic regulator(s)* remains challenging.

To validate these findings and explore the relevance for human cardiac fibrotic disease, we inspected whether the associations found in the rat were replicated in the human heart. We mapped the human ortholog gene co-expression networks to rat M1 and M2, to the corresponding human loci syntenic to the two associated rat loci displayed in Supplementary Figure 17. In the case of rat network M2 (*“Insulin signalling pathway”*), none of the human ortholog co-expression networks (i.e. DCM human co-expression networks with significant overrepresentation of human orthologs to the rat genes included in M2: Hs-M28, Hs-M39 and Hs-M44, see Fig 1a-c), yielded enough evidence of mapping to the syntenic human locus (median BF of the genes in the network > 100, the regulatory locus in rat chromosome 4q12, is syntenic to human chromosome 7q21.3, see Supplementary Methods).

Nevertheless, the signal associated with rat network M1 (*“*ECM network*”)* was replicated in human heart. These results are discussed and presented in the main text.

**SUPPLEMENTARY METHODS**

**1. Enrichment for cardiac fibroblast and myocyte**

*﻿Description of the cardiac data*. This RNA-Seq data set consists of primary mouse cardiac fibroblasts (n=2) and cardiomyocytes (n=2). Neonatal cardiomyocytes and cardiac fibroblasts were isolated from 0-1 day old C57bl/6 mice using the procedure described in ^3^. Cells were cultured at 37ºC for 48h in the presence of 10% fetal calf serum in DMEM medium. Total RNA was purified using the RNeasy kit (Qiagen) and sequenced with the Illumina Genome Analyzer IIx, 2 replicates per condition at 75 bp, paired-end library, one lane per replicate (sequencing depth of roughly 40M reads).

TopHat2 (version 2.0.13) ^4^ was used to align the paired-end reads to the GRCm38 mouse genome using available transcript annotations from Ensembl release 76. Mean insert size and standard deviation were computed empirically from uniquely mapping, perfect matching mate pairs via a preliminary alignment with Bowtie2 (version 2.2.3) ^5^. These were supplied as input parameters to TopHat2. Default parameterizations were used for the rest of options. Read counts for each gene were retrieved using HTSeq (version 0.6.1p1) with default parameters ^6^.

*Enrichment test.* When inferring gene co-expression networks from complex tissue (i.e. LV), the obtained gene networks could be capturing transcriptional programs coming from specific cell types. We set out to explore this by analyzing gene expression data from the two most abundant cell types in the heart: cardiac fibroblasts and myocytes ^7^. We ran a test to assess whether any of the human DCM networks were overrepresented for genes with higher or lower expression levels in cardiac fibroblasts when comparing to cardiomyocytes. More specifically, we carried out a GSEA analysis of cell type gene expression data from the mouse cardiac fibroblasts and myocytes described in the previous section. Mouse-human orthologs relationships were downloaded from Ensembl archive (Ensembl version GRCh37). Using this annotation, all mouse genes were mapped to their corresponding human ortholog by taking only those human genes with one-to-one (ortholog_one2one) human-mouse relationship. This resulted in 11,862 genes. The cardiac fibroblast-to-myocyte fold change was computed for each of these 11,862 genes by dividing the average expression counts in cardiac fibroblasts by the average expression counts in myocytes. This leads to a cardiac fibroblast-to-myocyte fold change value for each gene representing how many times is higher or lower the expression of that gene in fibroblasts in comparison to myocytes. Afterwards, all the human genes (11,862 genes) were ranked by their corresponding value of fibroblast-to-myocyte fold change. Then, this information was used as ranked list to run GSEA ^8^. In this GSEA analysis we used the human DCM clusters (considering only the genes present in these 11,862 genes) as gene sets. We run GSEA (version 2.1.0) ^8^ in classic, pre-ranked mode with 10,000 iterations by setting the maximum gene set size to 5,000 and the minimum gene set size to 10.

*Analysis of human networks in rTOF RV tissue.* To explore the conservation in rTOF RV patients of the prioritized human network inferred in human DCM LV, we implemented an empirical permutation test that assesses how likely is it to find by chance a set of genes of the same size as the network with a similar degree of average absolute correlation in the data set under testing. In this empirical we assess the conservation of each human network in rTOF expression data set as following: 1) From the background to test (i.e. expression data set in which we want to see whether our network is conserved), randomly sample without replacement a set of genes as the same size of the co-expression network under testing. Compute the mean absolute correlation of the sampled genes without considering self-correlations. We repeat this step for a number of times (e.g. 100,000) to generate a null distribution of simulated mean correlations. 2) Compute the mean absolute correlation (without considering self-correlations) of the genes in each network in the data set in which we are testing for conservation (rTOF). 3) Compute the corresponding empirical p-value by using the values obtained in steps 1 and 2 as in ^9^: P=(r+1) / (n+1), where n is the number of simulations (number of permutations) and r is the number of simulated mean correlation values higher than the actual mean correlation value of the network of interest. We ran this test using Turkey’s biweight midcorrelation and 100,000 permutations, which yield a minimum nominal significance level of 1x10^-5^. The p-value obtained for the test of the hECM-network in rTOF RV patients was 1x10^-5^. Results for all the prioritized networks are presented in Supplementary Fig. 15.

**2. Network enrichment for genes differentially expressed by TGFβ1 and TGFβ2.**

*Description of the cells.* Human ventricular cardiac fibroblasts (passage 5) were grown to ~90% confluence in 1% fetal bovine serum medium for 24h. Then, they were incubated in triplicates (9 samples in total) in 1% fetal bovine serum medium for 24h with +/-: human TGFβ1 (R&D cat100B, 5ng/ml), or human recombinant TGFβ2 (Millipore cat GF113, 5ng/ml). Supplier of ventricular human cardiac fibroblasts: Lonza (Catalogue number: CC-2904), product name: NHCF-V human cardiac fibroblasts-ventricular. Media kit: Fibroblast Growth Medium-3 BulletKit™ Kit. Catalogue number: CC-4526.

*RNA extraction, sequencing and RNA-seq data processing*. Total RNA was extracted and then sequenced with the Solexa platform (2 x 100 bp paired-end). Reads were mapped to the human genome (homo sapiens GRCh37.73, human genome 19 version) using TopHat 2.0.6 ^10^ and gene counts were computed with HTSeq 0.5.4p3 ^6^.

*Differential Expression analysis.* Raw human counts were used as input to run the R package DESeq2 1.6.3.^11^. DESeq R function was run, considering the following two treatments in the primary human cardiac fibroblasts data: 24h induction with TGFβ1 (n=3) and 24h induction with TGFβ2 (n=3). In both cases, as baseline, 24h control human cardiac fibroblasts (n=3) were used. Due to the low number of samples, to obtain the adjusted DESeq2 DE p-values, the outlier correction parameter *cooksCutoff* from the DESeq2 package function *results* was set to false. Results for the hECM-network genes can be found in Supplementary Table 3c.

*Enrichment test for 24hrs TGFβ DE genes.* In this analysis only genes expressed in human cardiac fibroblasts that were also robustly expressed in the DCM LV (i.e. WGCNA input set of genes) were considered for both the ranked list and human networks. Genes were ranked by the output statistic of the differential expression test (Wald statistic, as we were using default parameterizations). After this, GSEA was run twice using as gene set the co-expression networks built in human DCM LV, and as ranked list all the genes ranked by the statistic of the differential expression test after 24h induction with either TGFβ1 or with TGFβ2. GSEA 2.1.0 was run in classic (pre-ranked) mode with 10,000 iterations, setting maximum gene set size to 5,000 and minimum gene set size to 10.

*hECM-network enrichment in DCM***.** We carried out an enrichment analysis of the hECM-network by using the results of the study published by Burke *et al.* ^12^. In this study they use a mouse genetic model of DCM and perform RNA-seq and differentially expression analysis in both cardiomyocytes and non-myocytes from hearts of control mice, mice with DCM and heart failure. Two lists of non-myocyte differentially expressed genes were downloaded from the Supplementary Table 3 from the study published by Burke *et al.* (comparisons “non-myocyte DCM vs WT” and “non-myocyte HF vs WT”) ^12^. As this study was carried out in mouse, to test for overrepresentation of the hECM-network genes in these lists of differentially expressed genes, we first mapped the genes included in the hECM-network (683 human genes) to their one-to-one mouse orthologs that were also expressed in the mouse heart. For this we downloaded all the human-to-mouse one-to-one orthologs from Biomart and also only considered genes expressed in the mouse left ventricle with FPKM>1 in at least two mice (as described in section “Analysis of WWP2^Mut/Mut^ mouse RNA-seq data”). This resulted in a reduction of the 683 hECM-network genes to 415 genes as present in the network, expressed in the mouse heart left ventricle and with one-to-one human-mouse ortholog relationship. We then generated a background for this test composed by the set of mouse genes expressed in the mouse heart left ventricle also with one-to-one human-mouse ortholog relationship (10,271 genes). In the next step we selected only those genes in the differentially expressed gene lists provided by Burke *et al.* that were present in this 10,271 mouse gene background. Finally, we carried out a Fisher’s exact test testing for significant overlap between the hECM-network (now mapped to mouse, 415 genes) and the set of genes present in our 10,271 mouse gene background that were upregulated in non-cardiomyocytes in DCM and HF differentially expressed gene lists separately (2,005 and 2,162 genes respectively). We used R function used *fisher test* and we set the *alternative* parameter to *“greater”* (as we tested for overrepresentation). This analysis resulted in 226 genes present in the hECM-network (now mapped to mouse) that were upregulated in DCM in non-myocytes and 221 genes upregulated in HF in non-myocytes (these represent 54% and 53% of the 415 network genes).

*Enrichments of SMAD targets in the hECM-network*. The ChEA TF curated database of experimental TF DNA binding mammalian data ^13^ was queried for experimentally validated SMAD1-8 targets. SMAD targets available for SMAD1-4 were also coming from the following references ^14-17^.

Overrepresentation of SMAD targets within the hECM-network was assessed by Fisher's exact test. Steps followed: 1) Remove SMAD targets that were not robustly expressed in DCM heart (here we considered the background from where the DCM network were inferred, n=14,281 genes). By applying this filtering, the initial number of SMAD targets (SMAD1 = 610, SMAD2 = 1936, SMAD3 = 3233 and SMAD4 = 5012) got reduced to SMAD1 = 386, SMAD2 = 1396, SMAD3 = 2076 and SMAD4 = 3164. 2) For each SMAD1-4 a Fisher's exact test was computed with the R function *fisher.test* and setting the *alternative* parameter to *“greater”* (as we are testing for overrepresentation). The gene background used for computing the contingency table was the set of genes from which the human DCM clusters were inferred (robustly expressed in the LV human cohort n=14,281). The full list of SMAD targets downloaded including the ones detected in the hECM-network are included in Supplementary Table S3d.

**3. Bayesian network-expression QTL (network-eQTL) analysis: description of HESS runs.** HESS runs were performed with 20,000 sweeps, 5,000 burn ins and default parameterizations. From the output MPPIs, BFs were computed by using the formula described above. For each SNP, the interquantile range of the network genes BFs distribution was plotted by using the function errbar from the R package Hmisc 4.1-1. The resulting BFs are shown in Fig. 2a-c. In Fig. 2c the linkage disequilibrium (LD) block graph was computed from the LV genotyped samples (n=187) by using the R package snpStats with the *“R.squared”* LD measure. MPPIs and BFs of association between the regulatory SNP rs9936589 and the individual genes in the hECM-network (for both the DCM patients and controls runs) are also included in Supplementary Table 3a.

**Supplementary References**

1. Moreno-Moral, A. & Petretto, E. From integrative genomics to systems genetics in the rat to link genotypes to phenotypes. *Dis Model Mech* **9**, 1097-1110 (2016).

2. Robert, E.K. & Adrian, E.R. Bayes Factors. *Journal of the American Statistical Association* **90**, 773-795 (1995).

3. Brand, N.J., Lara-Pezzi, E., Rosenthal, N. & Barton, P.J. Analysis of cardiac myocyte biology in transgenic mice: a protocol for preparation of neonatal mouse cardiac myocyte cultures. *Methods Mol Biol* **633**, 113-124 (2010).

4. Kim, D.*, et al.* TopHat2: accurate alignment of transcriptomes in the presence of insertions, deletions and gene fusions. *Genome Biol* **14**, R36 (2013).

5. Langmead, B. & Salzberg, S.L. Fast gapped-read alignment with Bowtie 2. *Nat Methods* **9**, 357-359 (2012).

6. Anders, S., Pyl, P.T. & Huber, W. HTSeq--a Python framework to work with high-throughput sequencing data. *Bioinformatics* **31**, 166-169 (2015).

7. Souders, C.A., Bowers, S.L. & Baudino, T.A. Cardiac fibroblast: the renaissance cell. *Circ Res* **105**, 1164-1176 (2009).

8. Subramanian, A.*, et al.* Gene set enrichment analysis: a knowledge-based approach for interpreting genome-wide expression profiles. *Proc Natl Acad Sci U S A* **102**, 15545-15550 (2005).

9. North, B.V., Curtis, D. & Sham, P.C. A note on the calculation of empirical P values from Monte Carlo procedures. *Am J Hum Genet* **71**, 439-441 (2002).

10. Trapnell, C., Pachter, L. & Salzberg, S.L. TopHat: discovering splice junctions with RNA-Seq. *Bioinformatics* **25**, 1105-1111 (2009).

11. Love, M.I., Huber, W. & Anders, S. Moderated estimation of fold change and dispersion for RNA-seq data with DESeq2. *Genome Biol* **15**, 550 (2014).

12. Burke, M.A.*, et al.* Molecular profiling of dilated cardiomyopathy that progresses to heart failure. *JCI Insight* **1**(2016).

13. Lachmann, A.*, et al.* ChEA: transcription factor regulation inferred from integrating genome-wide ChIP-X experiments. *Bioinformatics* **26**, 2438-2444 (2010).

14. Chen, X.*, et al.* Integration of external signaling pathways with the core transcriptional network in embryonic stem cells. *Cell* **133**, 1106-1117 (2008).

15. Koinuma, D.*, et al.* Chromatin immunoprecipitation on microarray analysis of Smad2/3 binding sites reveals roles of ETS1 and TFAP2A in transforming growth factor beta signaling. *Mol Cell Biol* **29**, 172-186 (2009).

16. Kim, S.W.*, et al.* Chromatin and transcriptional signatures for Nodal signaling during endoderm formation in hESCs. *Dev Biol* **357**, 492-504 (2011).

17. Kennedy, B.A.*, et al.* ChIP-seq defined genome-wide map of TGFbeta/SMAD4 targets: implications with clinical outcome of ovarian cancer. *PLoS One* **6**, e22606 (2011).
