# Supplementary material for: WWP2 regulates pathological cardiac fibrosis by modulating SMAD2 signaling
Source: Nat Commun. 2019 Aug 9;10:3616. doi: 10.1038/s41467-019-11551-9 (PMC6689010; doi:10.1038/s41467-019-11551-9)
Supplement: Supplementary file 3 — Description of Additional Supplementary Files [file 41467_2019_11551_MOESM3_ESM.docx]

**Description of Supplementary Files**

**File Name:** Supplementary Data 1

**Description:** Rat data. a: Overview of the rat networks and association of the rat networks to fibrosis (GSEA analysis ranking the rat genes by Spearman’s ranked correlation p-value of association between gene expression levels and fibrosis measurements). b: Break down of the genes included in each network. c: Perivascular and interstitial fibrosis and mean blood pressure values in each rat RI strain.

**File Name:** Supplementary Data 2

**Description:** Human DCM co-expression networks. a: Summary of human DCM networks. b: Genes included in each DCM network.

**File Name:** Supplementary Data 3

**Description:** Annotation of human ECM-network and WWP2 information. a: Bayesian networkQTL HESS mapping (Marginal posterior probability of inclusion, MPPI and Bayes Factor, BF) results at the WWP2. regulatory SNP (rs9936589). Inf refers to infinitive. b: Genes in the hECM-network that are annotated in Gene Ontology Cellular Component (CC) database as ("extracellular region"). c: Differential expression results for the genes included in the hECM-network in human fibroblasts after TGFB1 and TGFB2 24h stimulation (two comparison: TGFB1 (24h) versus control and TGFB1 (24h) versus control). NA refers to gene not expressed in human cardiac fibroblasts or gene that was detected as outlier by DESeq2. d: List of SMAD1-4 targets tested and genes in the hECM-network that are SMAD targets. e: Correlation of hECM-network genes with WWP2 in DCM and rTOF patients. f: Expression levels of all the WWP2 isoforms detected in the DCM patients.

**File Name:** Supplementary Data 4

**Description:** Pathway analysis in WWP2Mut/Mut mice. a: Functional gene set enrichment analysis (GSEA) results for the databases Hallmark, Reactome and Gene Ontology in the RNA-seq differential expression (DE) analysis between WWP2 loss of function mouse with chronic Angiotensin II infusion compared against the WT mouse with chronic Angiotensin II infusion. GSEA was computed ranking all genes by DESeq2 R package Wald test statistic. NES denotes normalized enrichment score. "P or N enrichment" denotes whether the gene set is upregulated, P (positive), or downregulated, N (negative). Terms associated with SMAD regulation are highlighted (bold). b: Same test as in a but instead of assessing for enrichment of pathways and gene sets from known databases, assessing for all the human co-expression networks (including the hECM-network).
