# Supplementary material for: WWP2 regulates pathological cardiac fibrosis by modulating SMAD2 signaling
Source: Nat Commun. 2019 Aug 9;10:3616. doi: 10.1038/s41467-019-11551-9 (PMC6689010; doi:10.1038/s41467-019-11551-9)
Supplement: Supplementary file 8 — Reporting Summary [file 41467_2019_11551_MOESM8_ESM.pdf]

nature research

Corresponding author(s): Enrico Petretto

Last updated by author(s): Jul 18, 2019

Reporting Summary

Nature Research wishes to improve the reproducibility of the work that we publish. This form provides structure for consistency and transparency in reporting. For further information on Nature Research policies, see Authors & Referees and the Editorial Policy Checklist.

Statistics

For all statistical analyses, confirm that the following items are present in the figure legend, table legend, main text, or Methods section.

n/a	Confirmed	
☐	☒	The exact sample size (*n*) for each experimental group/condition, given as a discrete number and unit of measurement
☐	☒	A statement on whether measurements were taken from distinct samples or whether the same sample was measured repeatedly
☐	☒	The statistical test(s) used AND whether they are one- or two-sided<br>*Only common tests should be described solely by name; describe more complex techniques in the Methods section.*
☐	☒	A description of all covariates tested
☐	☒	A description of any assumptions or corrections, such as tests of normality and adjustment for multiple comparisons
☐	☒	A full description of the statistical parameters including central tendency (e.g. means) or other basic estimates (e.g. regression coefficient) AND variation (e.g. standard deviation) or associated estimates of uncertainty (e.g. confidence intervals)
☐	☒	For null hypothesis testing, the test statistic (e.g. *F*, *t*, *r*) with confidence intervals, effect sizes, degrees of freedom and *P* value noted<br>*Give P values as exact values whenever suitable.*
☐	☒	For Bayesian analysis, information on the choice of priors and Markov chain Monte Carlo settings
☒	☐	For hierarchical and complex designs, identification of the appropriate level for tests and full reporting of outcomes
☐	☒	Estimates of effect sizes (e.g. Cohen's *d*, Pearson's *r*), indicating how they were calculated

Our web collection on statistics for biologists contains articles on many of the points above.

Software and code

Policy information about availability of computer code

Data collection	STAR 2.5.2b ; RSEM 1.2.31; TopHat 2.0.12; Bowtie2 2.2.3; Samtools 0.1.18; HTSeq 0.6.1;
Data analysis	DESeq2 1.6.3; flashClust 1.01-2; SNAP 2.2; WGCNA 1.42; DAVID Web Service 1.4.0; GSEA 2.1.0; HESS software (available at http://www.mrc-bsu.cam.ac.uk/software/); Hmisc 4.1-1; Cell Ranger 2.1.1; scran 1.8.4 ; scater 1.8.4; GSEA 2-2.2.2

For manuscripts utilizing custom algorithms or software that are central to the research but not yet described in published literature, software must be made available to editors/reviewers. We strongly encourage code deposition in a community repository (e.g. GitHub). See the Nature Research guidelines for submitting code & software for further information.

Data

Policy information about availability of data

All manuscripts must include a data availability statement. This statement should provide the following information, where applicable:

- Accession codes, unique identifiers, or web links for publicly available datasets
- A list of figures that have associated raw data
- A description of any restrictions on data availability

All the data generated in this study supporting the main findings have been deposited to NCBI's Gene Expression Omnibus (GEO) and accessible through GEO Series accession number GSE133017 [https://www.ncbi.nlm.nih.gov/geo/query/acc.cgi?acc=GSE133017], including GSE130468 [https://www.ncbi.nlm.nih.gov/geo/query/acc.cgi?acc=GSE130468] (bulk RNA-seq data from mouse heart) and GSE133015 [https://www.ncbi.nlm.nih.gov/geo/query/acc.cgi?acc=GSE133015] (single-cell RNA-seq data from mouse heart). The rest of the data are available from the authors on reasonable request, please refer to author contributions for specific data sets.

Field-specific reporting

Please select the one below that is the best fit for your research. If you are not sure, read the appropriate sections before making your selection.

☒ Life sciences ☐ Behavioural & social sciences ☐ Ecological, evolutionary & environmental sciences

For a reference copy of the document with all sections, see nature.com/documents/nr-reporting-summary-flat.pdf

Life sciences study design

All studies must disclose on these points even when the disclosure is negative.

Sample size	In the WT versus loss of function mice comparisons sample sizes were allocated as suggested in https://www.ncbi.nlm.nih.gov/pmc/articles/PMC4201821/ . Sample size of mice groups were determined by power analysis and n>=8 per group were used to account for the inherent variability.
Data exclusions	No data was excluded from the analyses apart from 2 DCM patients and 14 controls for quality control purposes (outlier samples).
Replication	All attempts were replicated, and the number of repeats are stated in figure legends.
Randomization	When comparing mice with different genotypes, littermate mice were assigned to the WT and Mut/Mut groups according to the results of genotyping (Supplementary Figure S2) and mice with the same genotype were randomly assigned to the control, AngII infusion or MI group using a simple random-sampling approach.
Blinding	The experimenters were blinded to the grouping information.

Reporting for specific materials, systems and methods

We require information from authors about some types of materials, experimental systems and methods used in many studies. Here, indicate whether each material, system or method listed is relevant to your study. If you are not sure if a list item applies to your research, read the appropriate section before selecting a response.

Materials & experimental systems

n/a	Involved in the study	
☐	☒	Antibodies
☐	☒	Eukaryotic cell lines
☒	☐	Palaeontology
☐	☒	Animals and other organisms
☐	☒	Human research participants
☒	☐	Clinical data

Methods

n/a	Involved in the study	
☒	☐	ChIP-seq
☒	☐	Flow cytometry
☒	☐	MRI-based neuroimaging

Antibodies

Antibodies used	Primary antibodies for immunofluorescence staining are anti-ACTA2 (Sigma-Aldrich, #A5228; 1:100), anti-S100A4 (Abcam, #ab41532, 1:100), anti-WWP2-FL/N (Santa Cruz biotechnology, #sc30052, 1:100), anti-Vimentin(Abcam, #ab45939, 1:100) and anti-FLAG(Sigma-Aldrich, #F7425, 1:100). Bovine Anti Rabbit IgG-CFL 488 (Santacruz Biotechnology, #sc-362260; 1: 50) and Bovine Anti Mouse IgG-CFL 488 (Santacruz Biotechnology, #sc-362256; 1:50) were used as secondary antibodies for immunofluorescence. Rhodamine Wheat Germ Agglutinin (WGA, Vector laboratories, #RL-1022) was used to stain the myocytes. VectaShield Mounting Medium (Vector laboratories, #H-1200) with DAPI was used to stain the nuclei and the slides were covered by coverslip.<br>Primary antibodies for WB are anti-WWP2 targeting N-terminal region (Santa Cruz biotechnology, #sc30052,1:500), anti-WWP2 targeting C-terminal region (Aviva Systems Biology, #ARP43089_P050,1:500), anti-ACTA2 (Sigma-Aldrich, #A5228,1:10,000), anti-S100A4 (Abcam, #ab41532, 1:500), anti-Vimentin (Abcam, #ab45939,1:500), anti-Periostin (Novus bio, # NBP1-30042, 1:500), anti-Fibronectin (Sigma, #SAB4500974, 1:500), anti-collagen 1 (Abcam, ab6308, 1:500) anti-pSMAD2 (CST, #18338,1:500), anti-SMAD 2/3 (CST, #3102, 1:500), anti-SMAD-4 (Santa Cruz biotechnology, #sc-7966, 1:500), anti-Ubiquitin (CST, #3933, 1:500), anti-FLAG (Sigma-Aldrich, #F7425, 1:1000). Loading control was blotted with anti-tubulin (Sigma-Aldrich, #T5168, 1:5000) and anti-GAPDH (Abcam, #ab8245, 1:5000). Anti-Lamin (abcam, #ab8984, 1:5000) and anti-PARP (abcam, #ab6079,1:5000) were used as nuclear controls. Blots were visualized by labeling with anti-Rabbit HRP (Bethyl laboratories, #A120-101P, 1:5000 or Thermo Fisher # 101023, 1:1000) and anti-Mouse HRP (Bethyl laboratories, #A90-116P, 1:5000).
Validation	Validation information can be obtained from the website of the antibody companies.

Eukaryotic cell lines

Policy information about cell lines

Cell line source(s)	Human cardiac fibroblasts: Lonza (Catalogue number: CC-2904), product name: NHCF-V human cardiac fibroblasts-ventricular. Media kit: Fibroblast Growth Medium-3 BulletKit™ Kit. Catalogue number: CC-4526. C2C12 mouse myoblast cell line, gifted by Dr. Lisa Tucker Kelloggs' laboratory (stated in methods).
Authentication	None of the cell lines were authenticated.
Mycoplasma contamination	Cell lines were negative for Mycoplasma contamination.
Commonly misidentified lines (See ICLAC register)	None used.

Animals and other organisms

Policy information about studies involving animals; ARRIVE guidelines recommended for reporting animal research

Laboratory animals	WWP2 mut/wt mice were generated based on C57BL/6J in the laboratory of Dr. Weiping Yu at Agency for Science, Technology and Research (A*STAR). Male littermate mice (8-12 weeks) were assigned to the WT and Mut/Mut groups according to the results of genotyping.
Wild animals	The study did not involve wild animals.
Field-collected samples	The study did not involve field-collected samples.
Ethics oversight	All relevant ethical regulations according to guidelines was issued by the National Advisory Committee on Laboratory Animal Research. Protocol with IACUC number 2016/SHS/1170 was approved by Institutional Animal Care and Use Committee of National University of Singapore, Duke-NUS Medical School.

Note that full information on the approval of the study protocol must also be provided in the manuscript.

Human research participants

Policy information about studies involving human research participants

Population characteristics	A subset of the DCM and control LV samples from the CEU population (96 DCM samples and 91 control samples, and the detail is listed in Heinig, M., et al. Natural genetic variation of the cardiac transcriptome in non-diseased donors and patients with dilated cardiomyopathy. Genome Biol 18, 170 (2017)) Tetralogy of Fallot (TOF) patients were under the care of the Adult Congenital Heart Disease service at the Royal Brompton Hospital, UK.
Recruitment	Repaired TOF patients were under the care of the Adult Congenital Heart Disease service at the Royal Brompton Hospital, UK. Right ventricle myocardial biopsies were made available via the Cardiovascular Biomedical Research Unit Biobank of the Royal Brompton & Harefield NHS Foundation Trust.<br>Human cardiac fibroblasts were isolated from right atrium (RA) appendage obtained from patients on cardiopulmonary-by-pass during cardiac surgery operations by digesting the tissue with Collagenase II.
Ethics oversight	The study on DCM patients was done in compliance with ethical regulation and was approved by the UK National Research Ethic Service (NRES) Committee London-Fulham. All patients gave informed consents.<br>All eligible patients of rTOF were under the care of the Adult Congenital Heart Disease service at the Royal Brompton Hospital, UK. . The Royal Brompton & Harefield NHS Trust and National Heart & Lung Institute Ethics Committee approved this protocol, with informed consent obtained from all study participants.

Note that full information on the approval of the study protocol must also be provided in the manuscript.
