# Supplementary material for: WWP2 regulates pathological cardiac fibrosis by modulating SMAD2 signaling
Source: Nat Commun. 2019 Aug 9;10:3616. doi: 10.1038/s41467-019-11551-9 (PMC6689010; doi:10.1038/s41467-019-11551-9)

Supplementary information for WB

WWP2 regulates pathological cardiac fibrosis by modulating SMAD2 signaling

Huimei Chen*, Aida Moreno-Moral*, et al.

Original blot of Figure 3

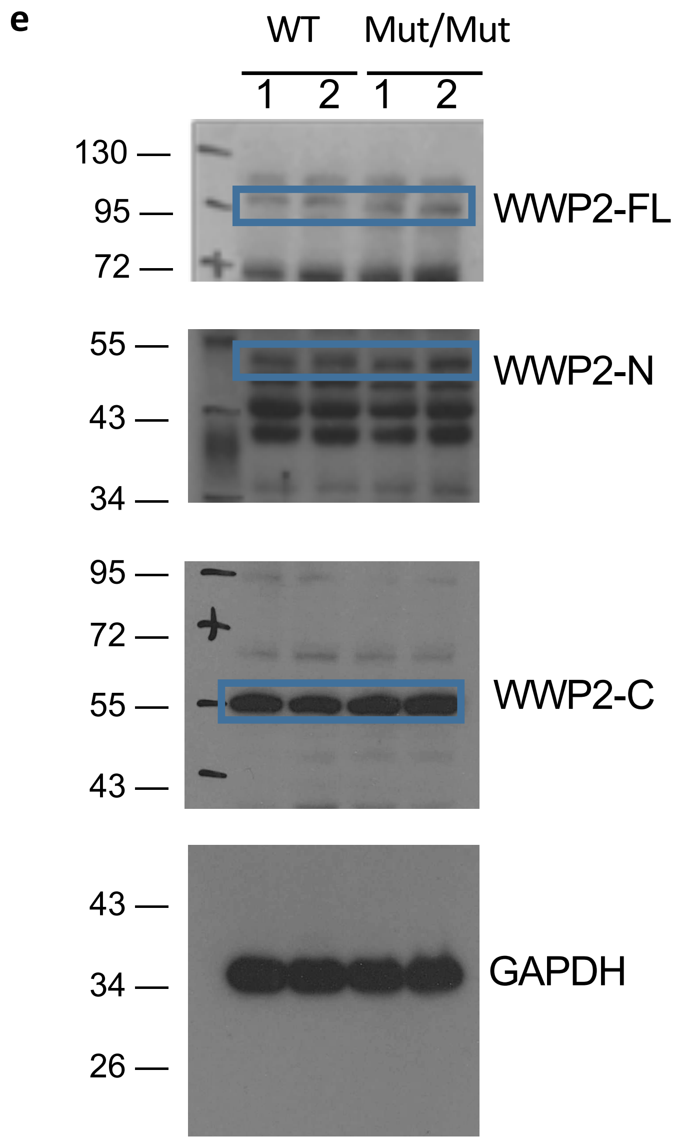

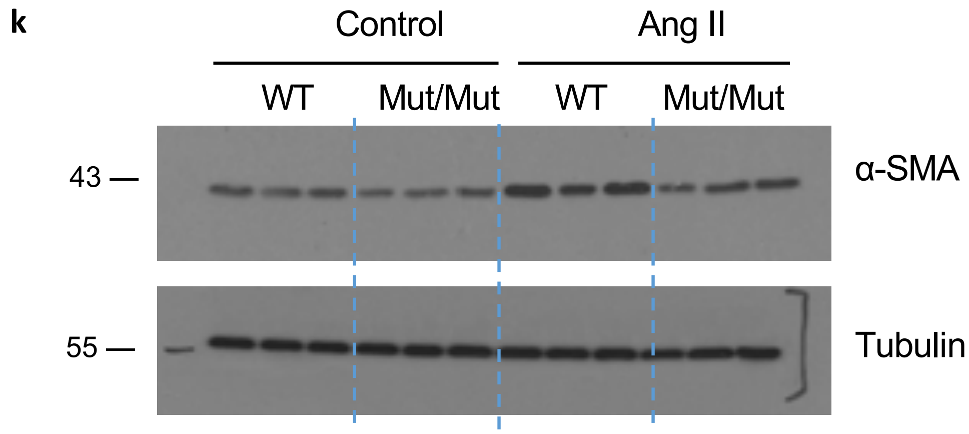

Original blot of Figure 3

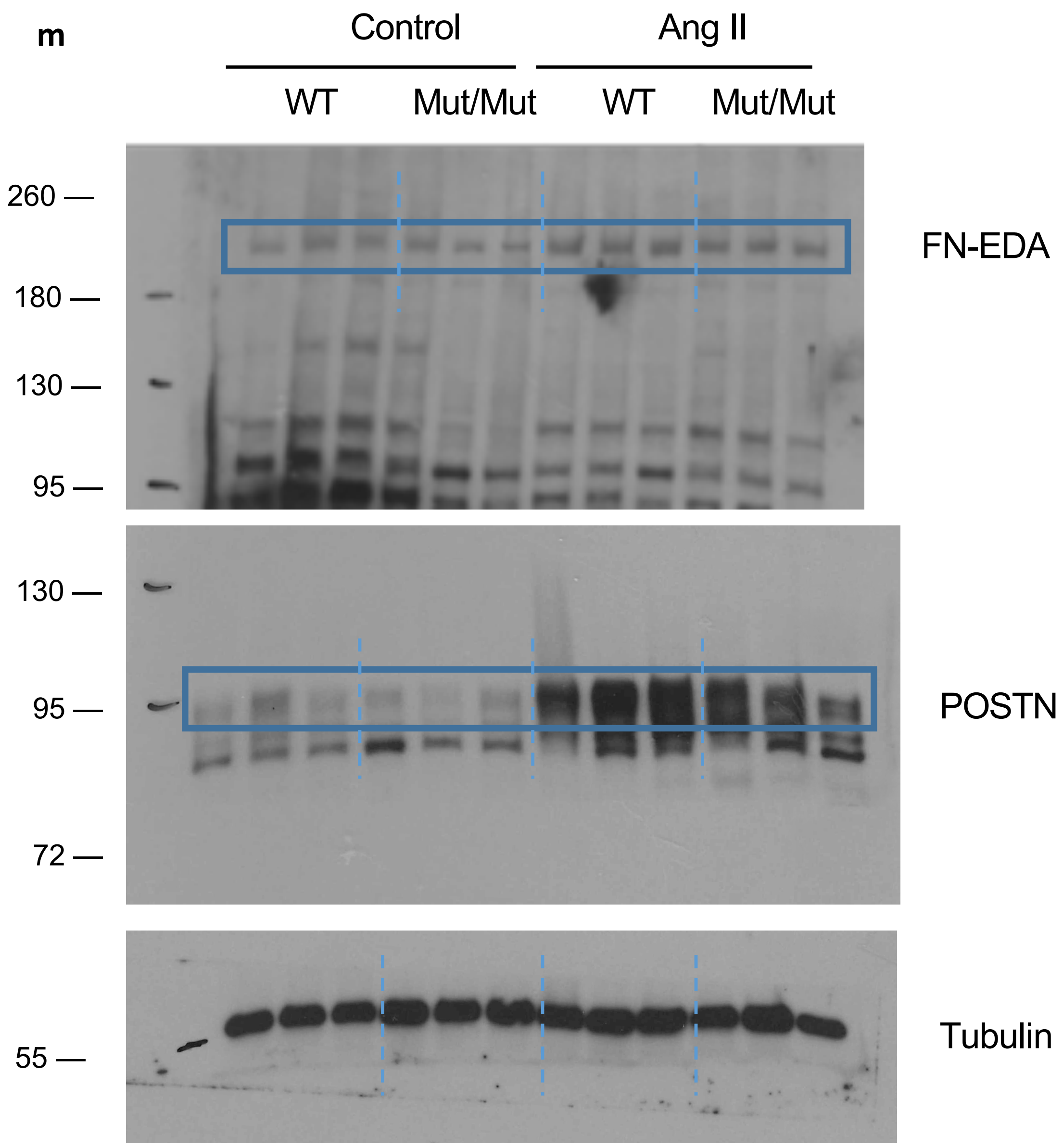

Original blot of Figure 4

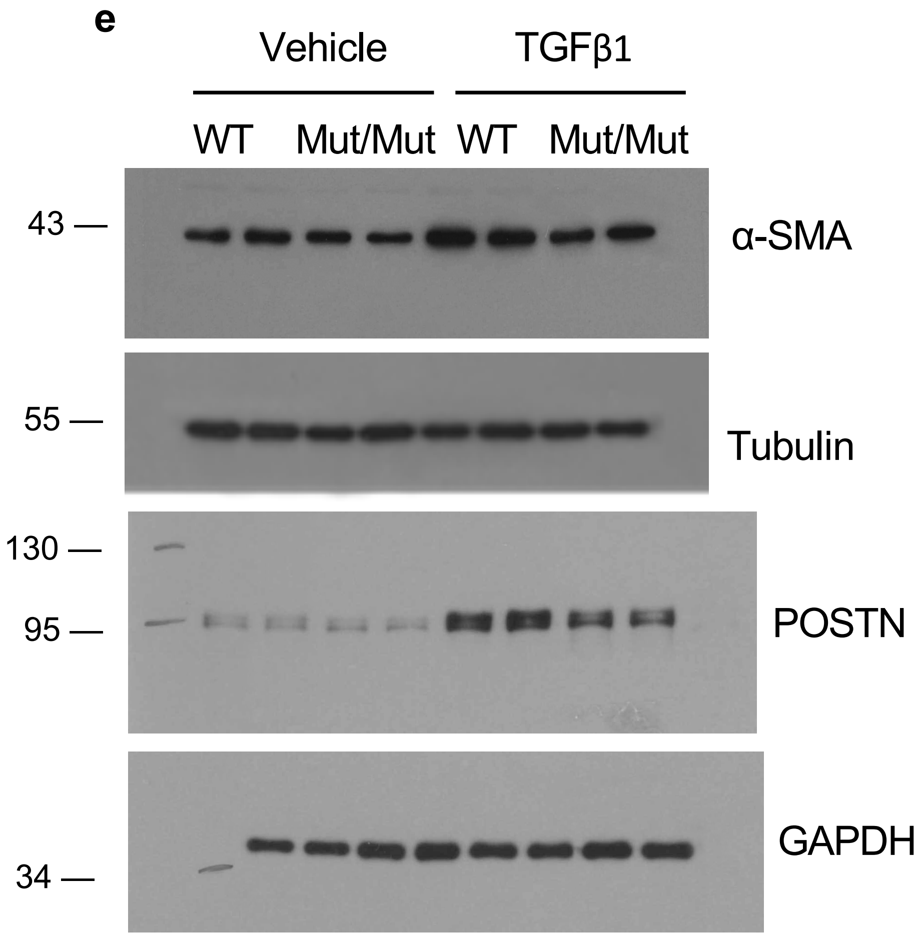

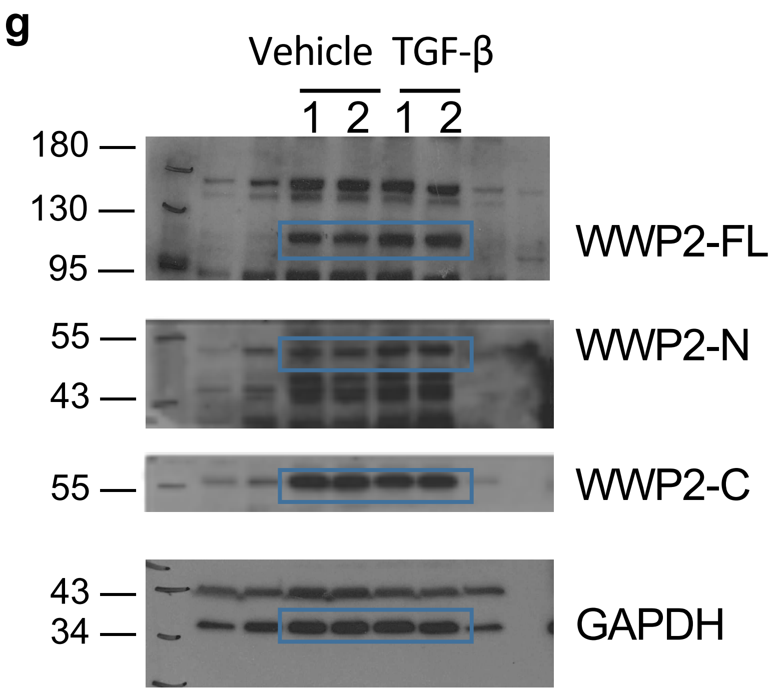

Original blot of Figure 4

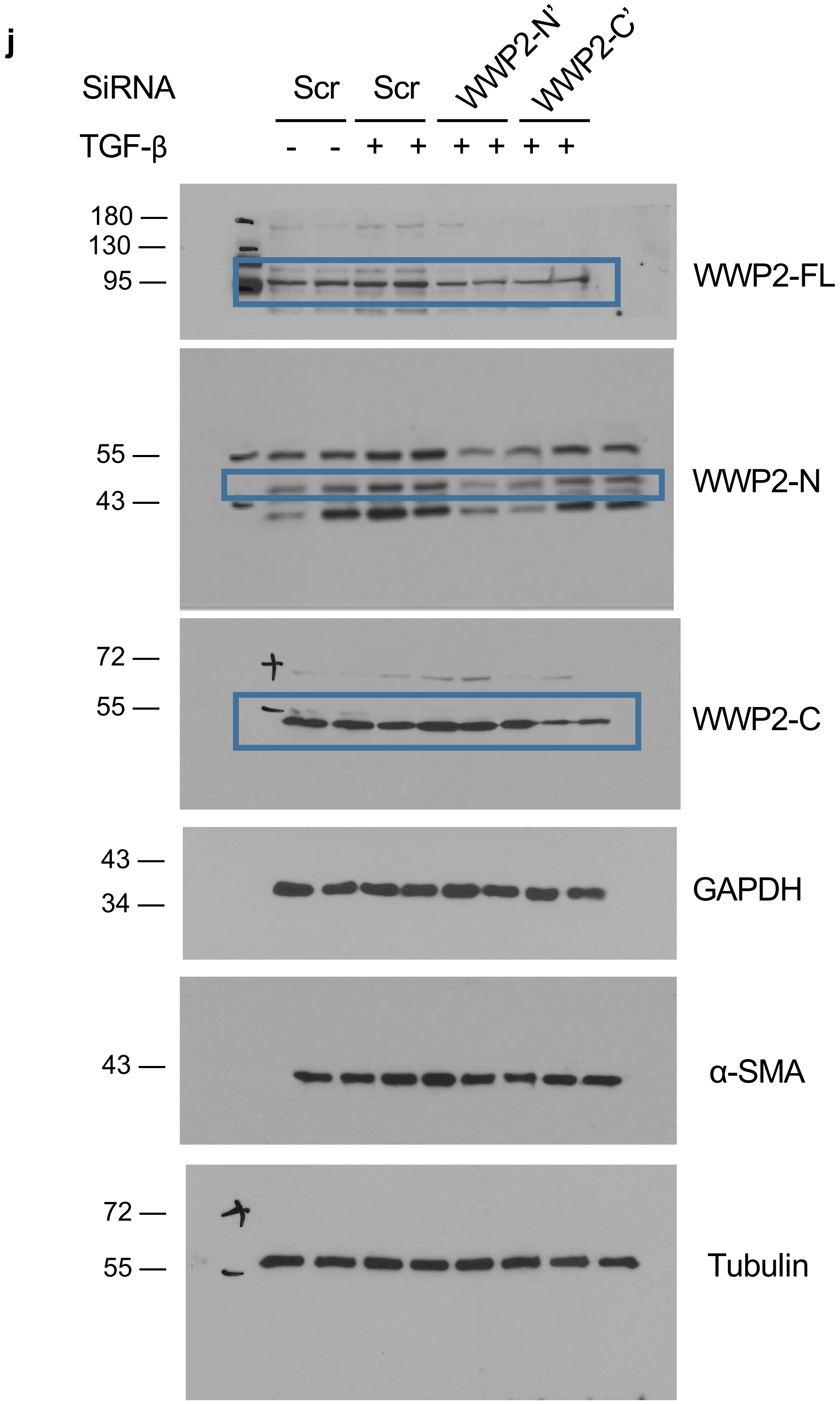

Original blot of Figure 4

n

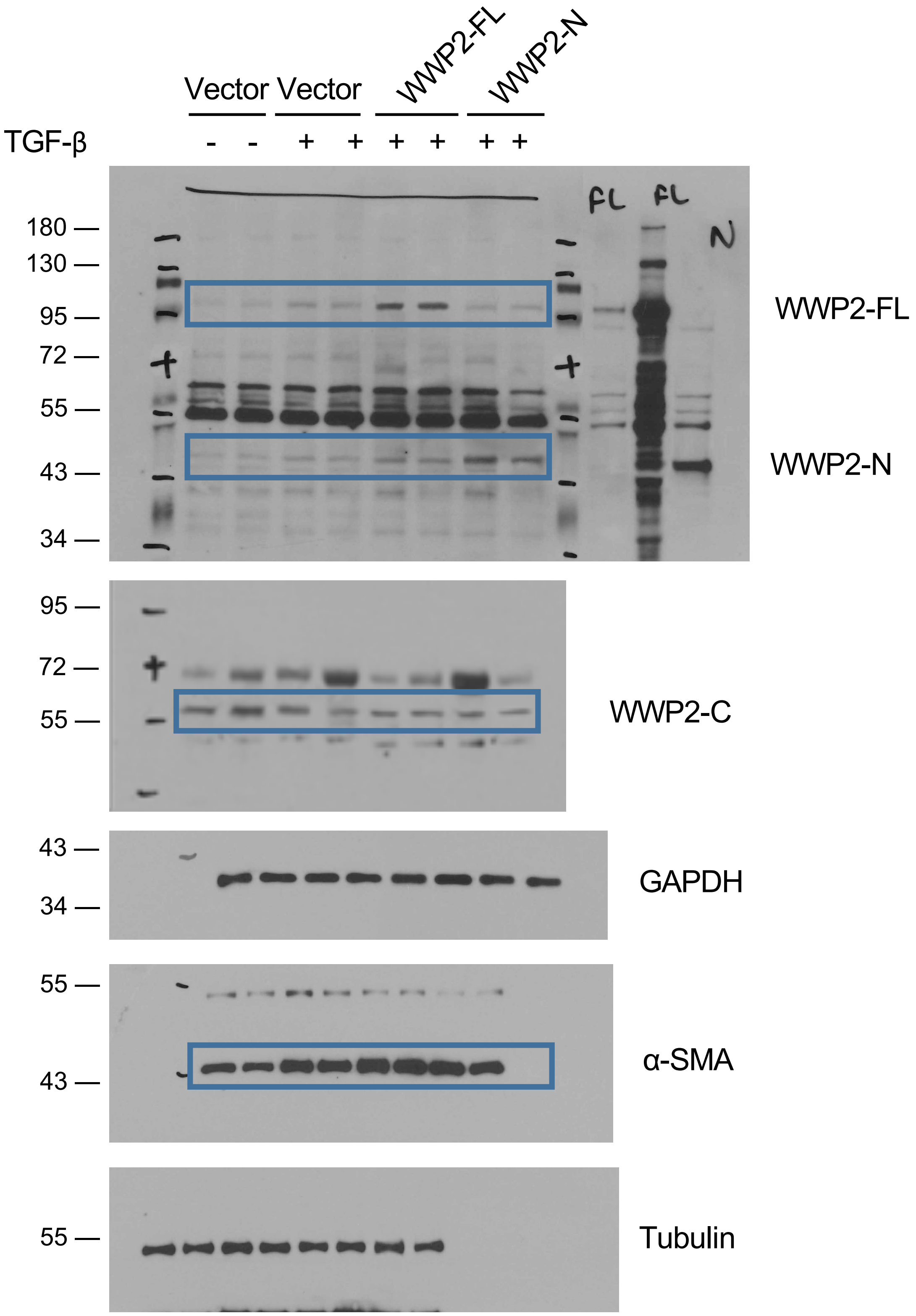

Original blot of Figure 5

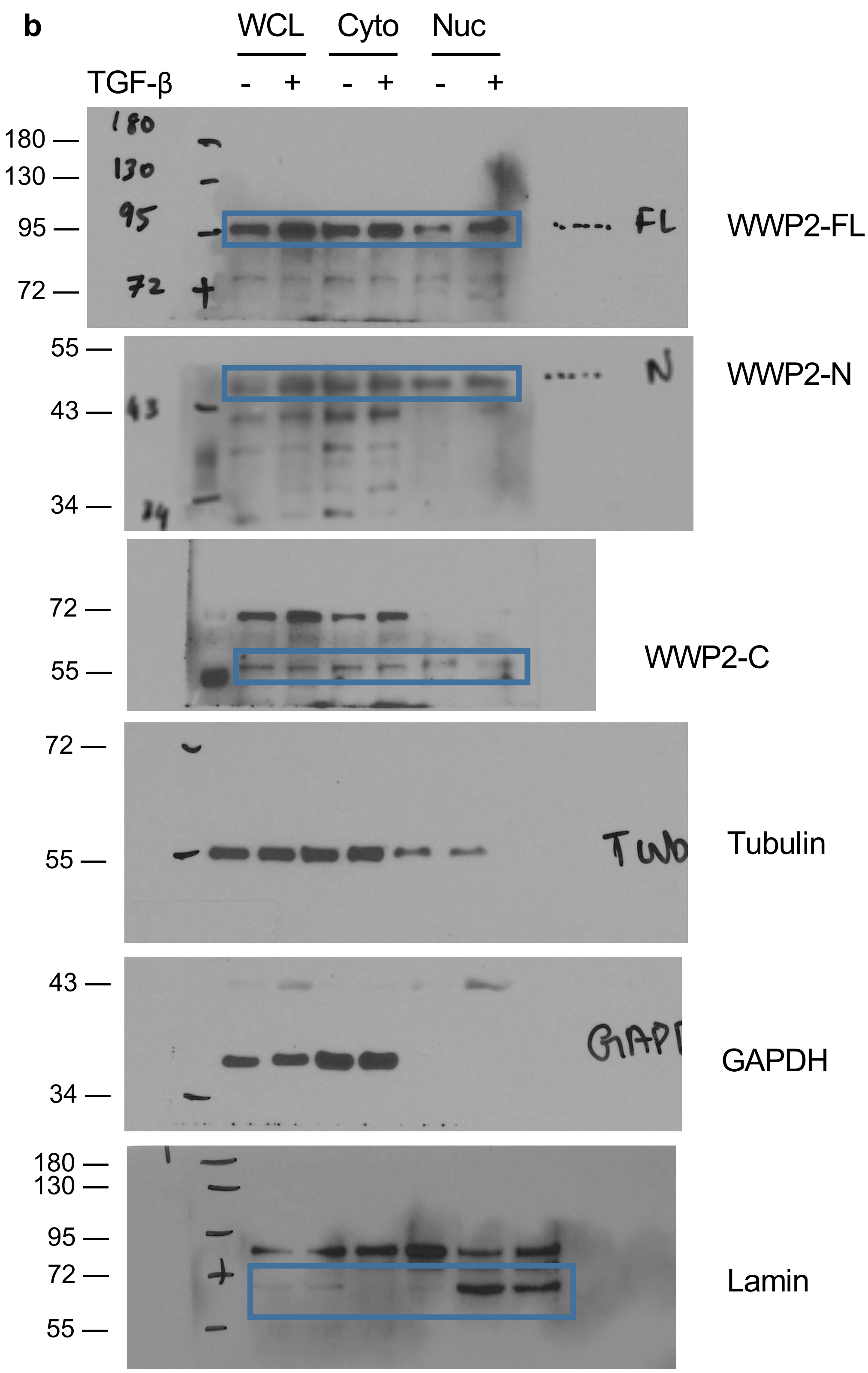

Original blot of Figure 5

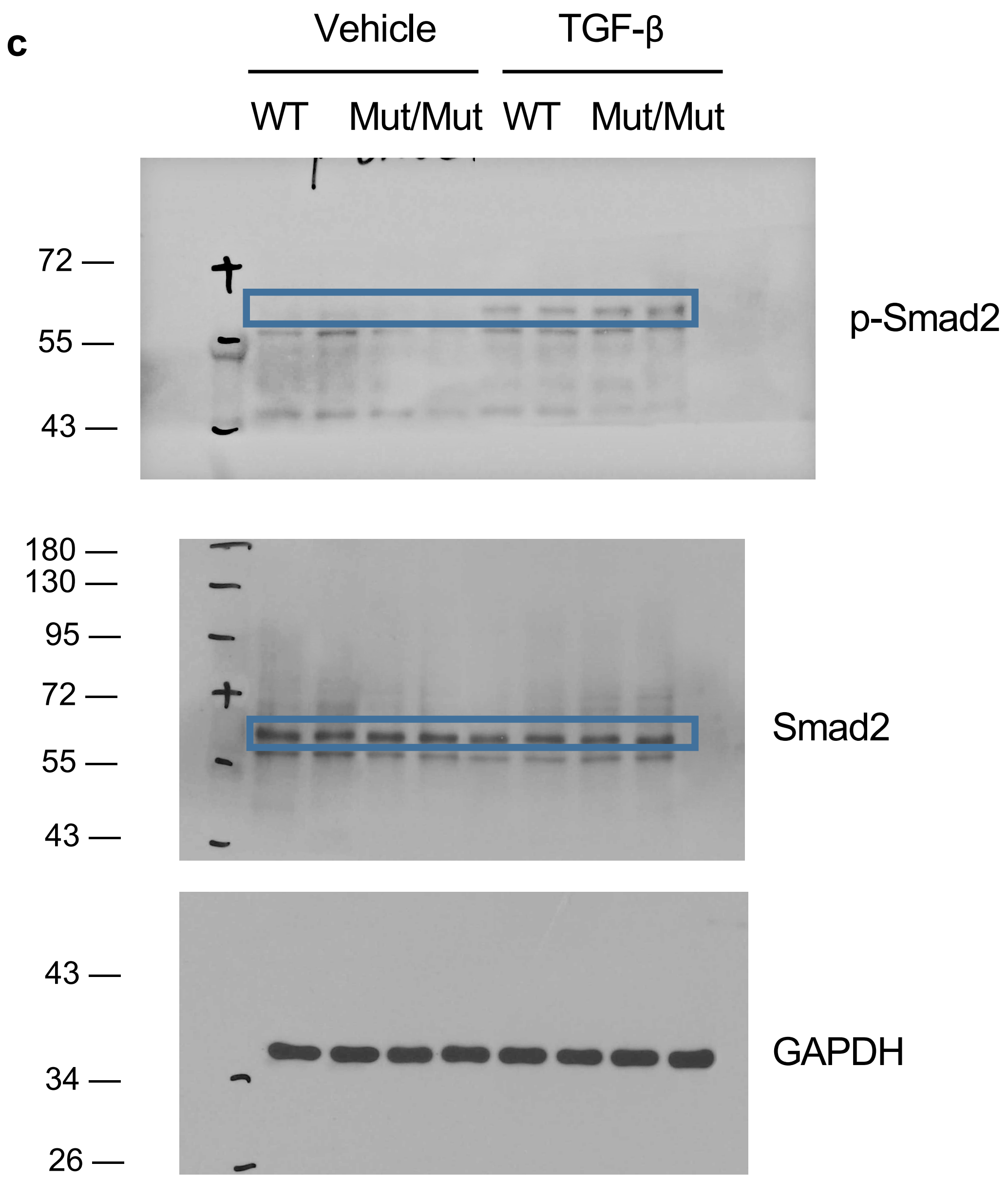

Original blot of Figure 5

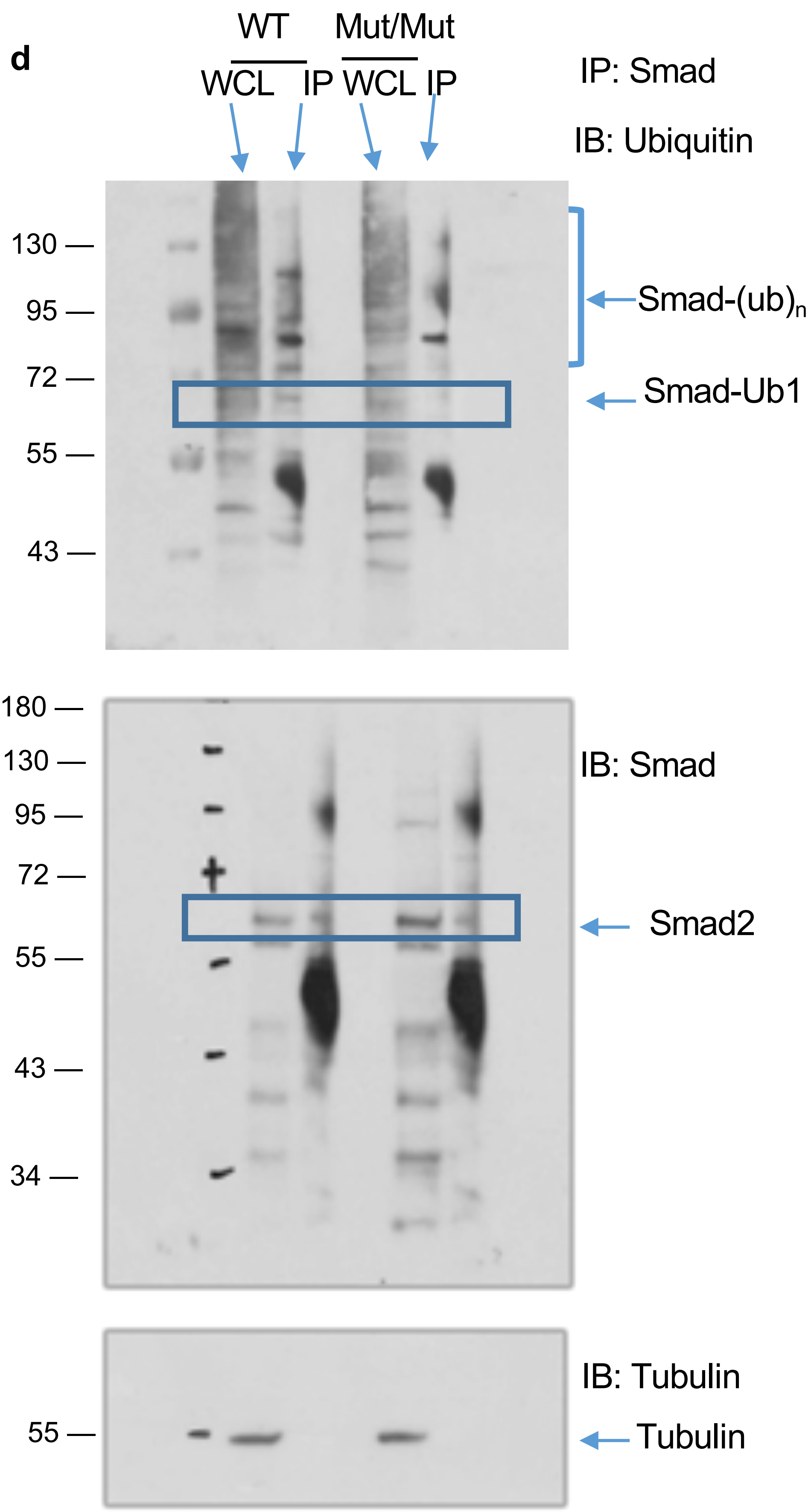

Original blot of Figure 5

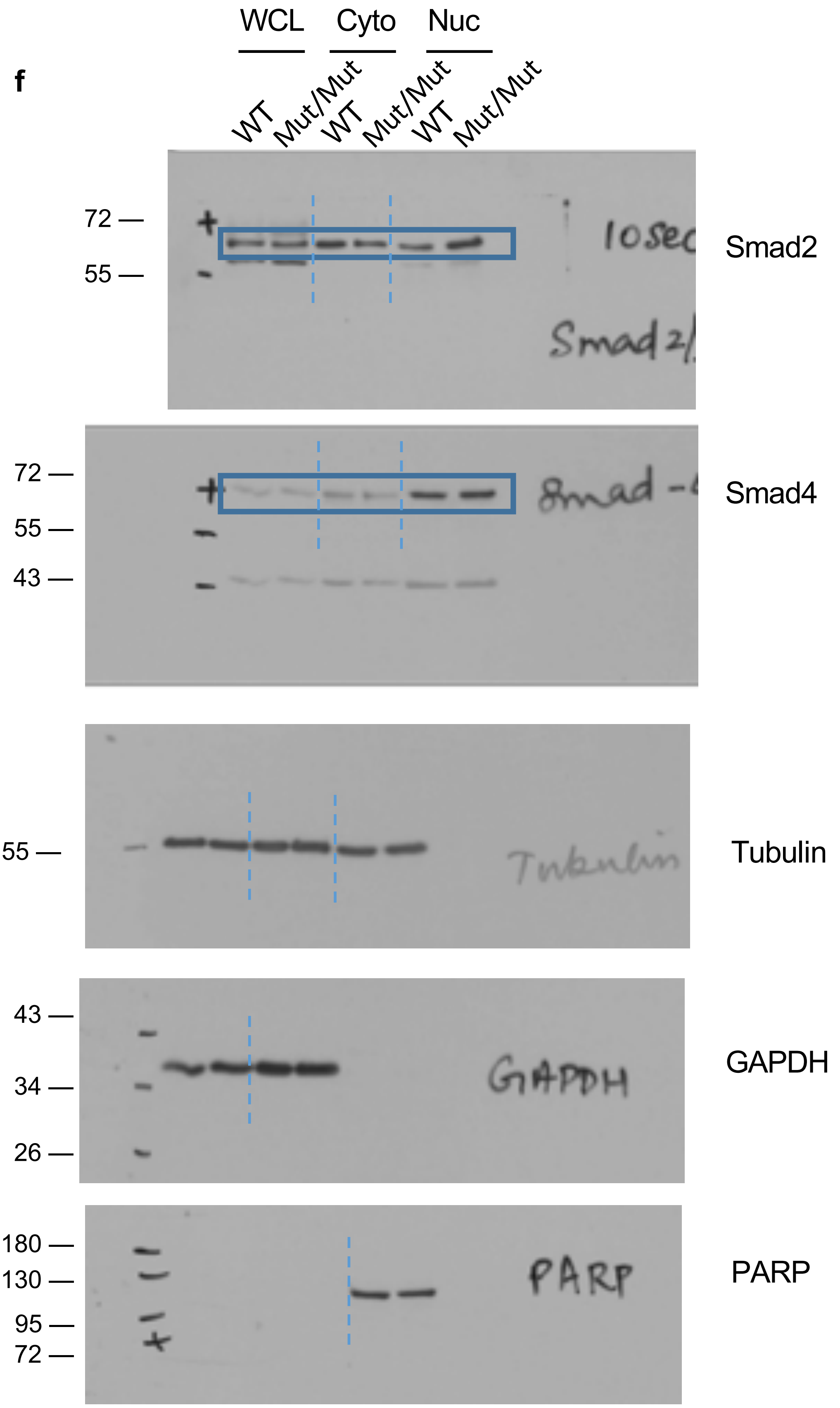

Original blot of Supplementary Figure 2

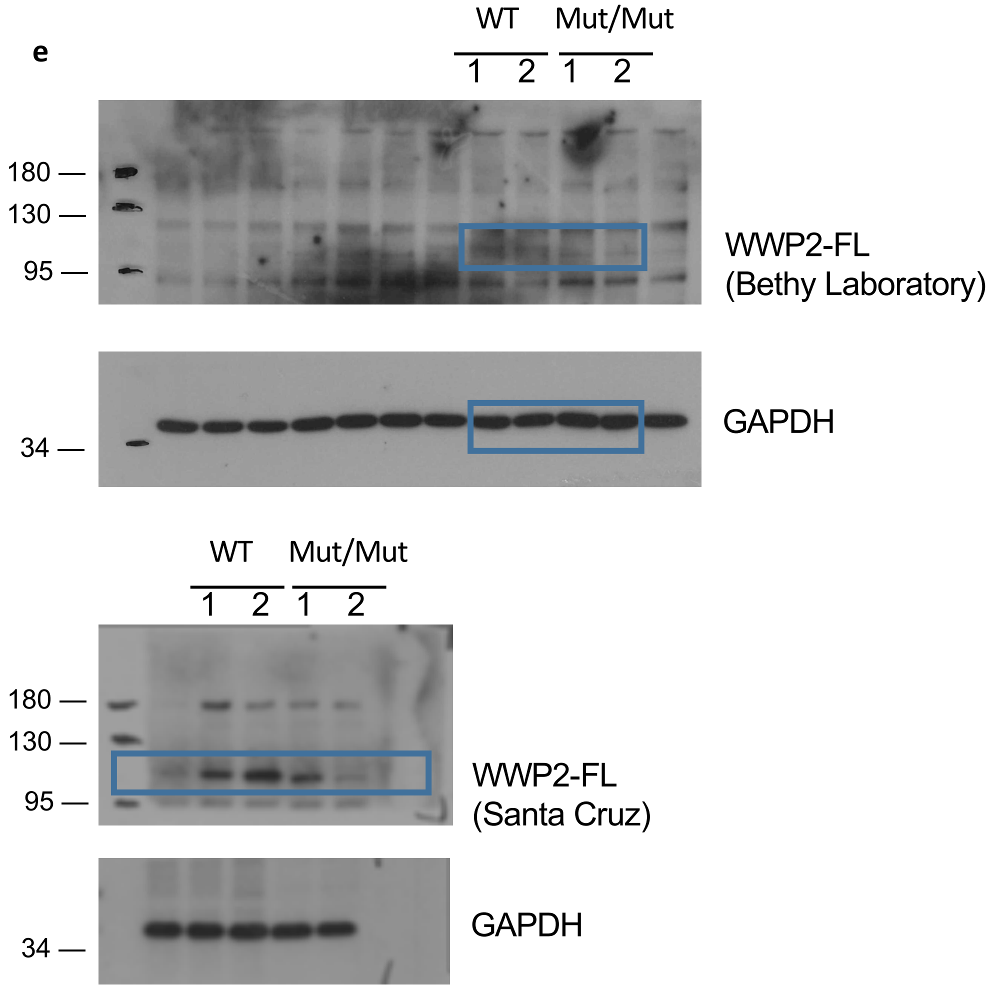

Original blot of Supplementary Figure 3

g

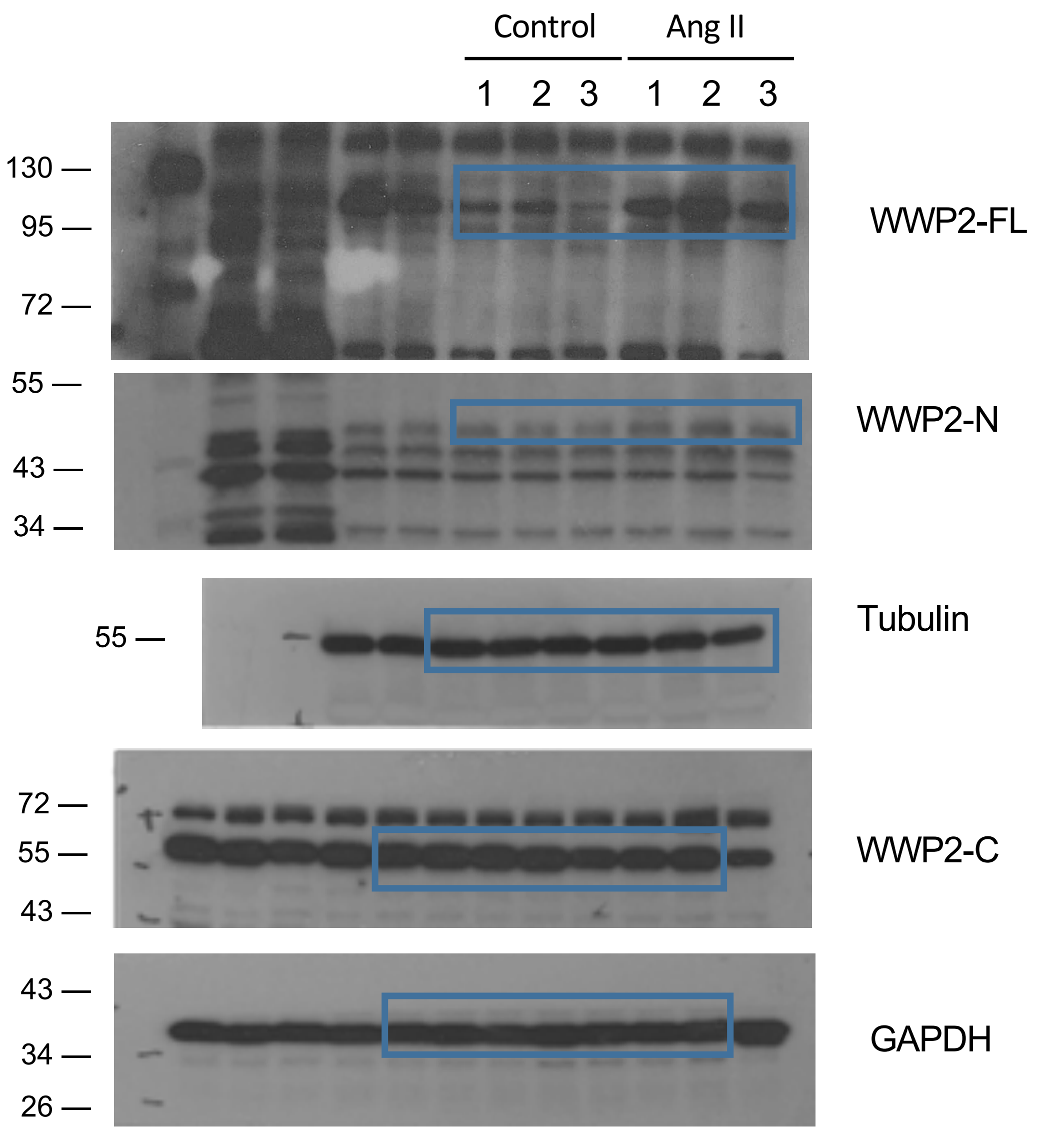

Original blot of Supplementary Figure 8

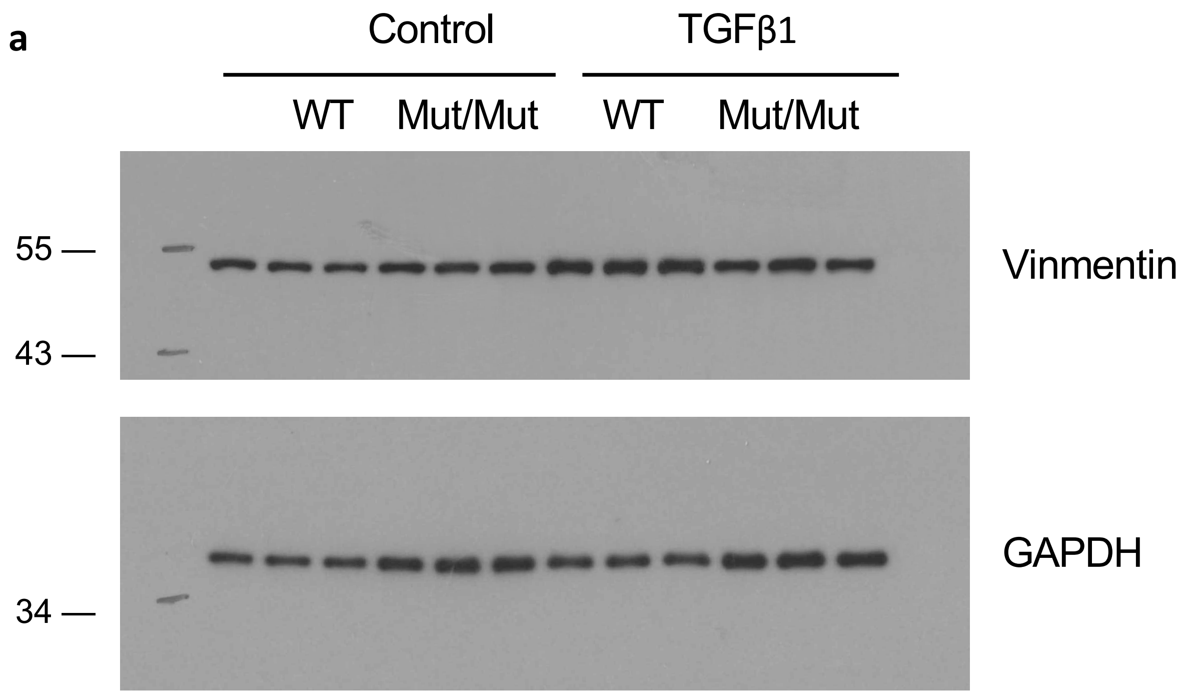

Original blot of Supplementary Figure 11

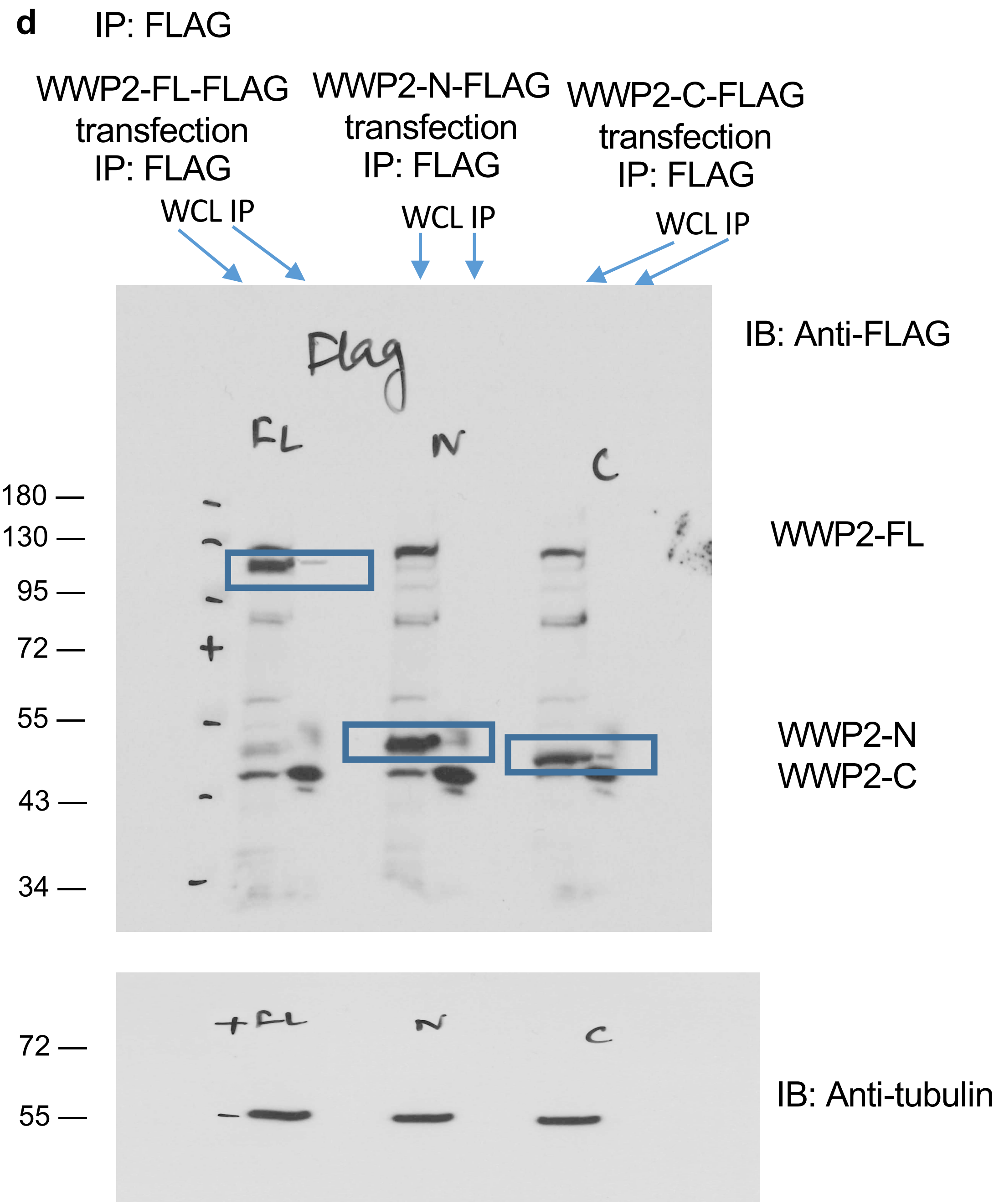

Original blot of Supplementary Figure 11

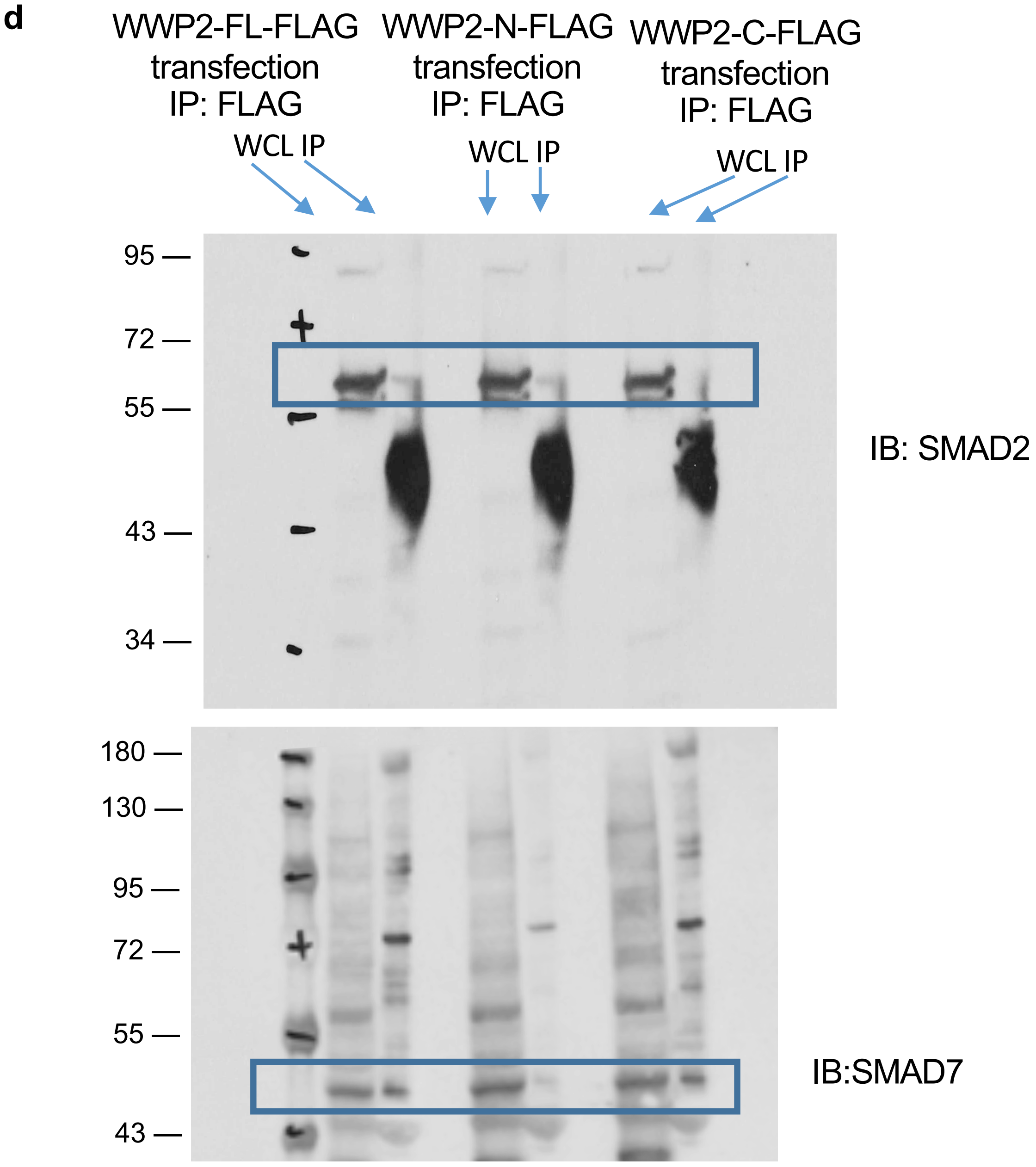

Original blot of Supplementary Figure 11

e

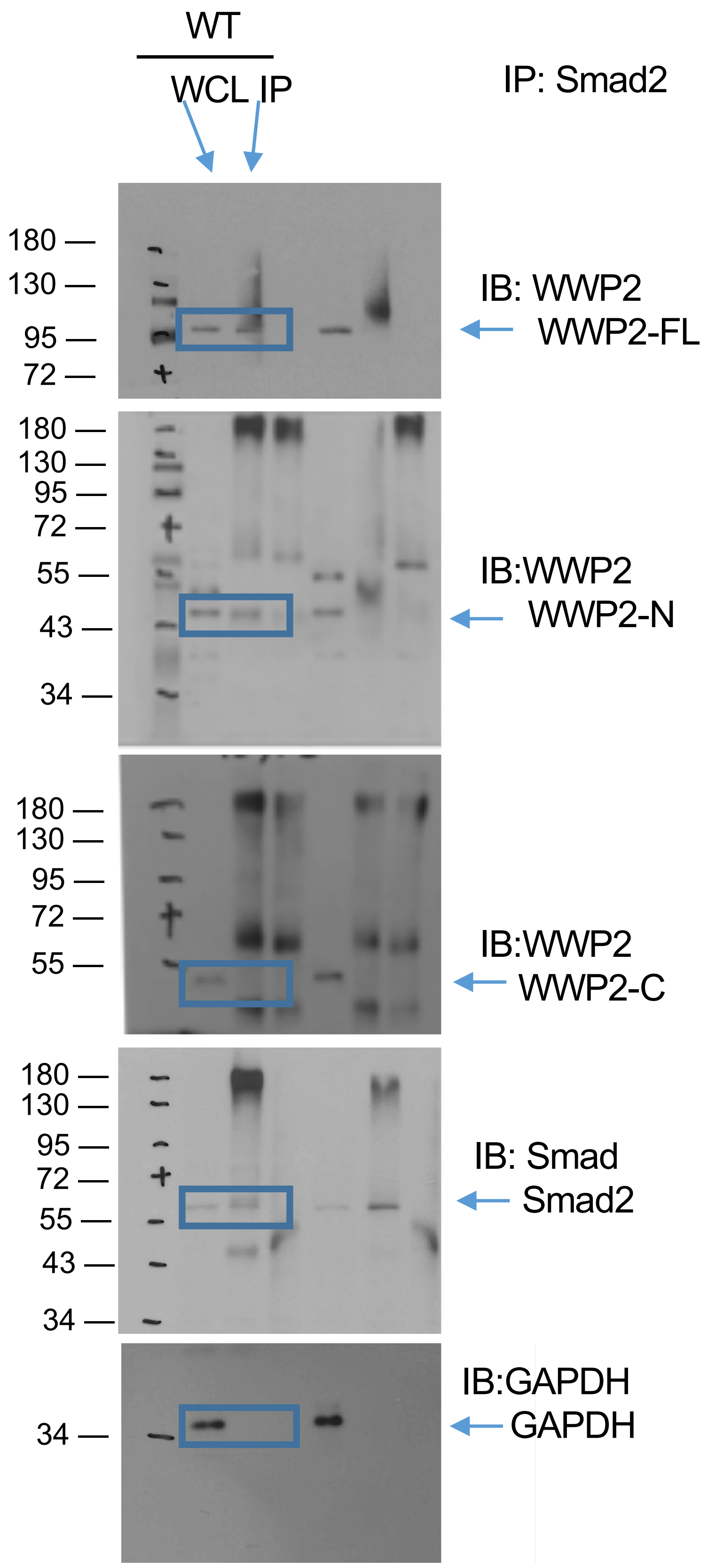
WT
WCL IP
IP: Smad2
180
130
95
72
IB: WWP2
WWP2-FL
180
130
95
72
55
43
34
IB:WWP2
WWP2-N
180
130
95
72
55
IB:WWP2
WWP2-C
180
130
95
72
55
43
34
IB: Smad
Smad2
34
IB:GAPDH
GAPDH

Original blot of Supplementary Figure 11

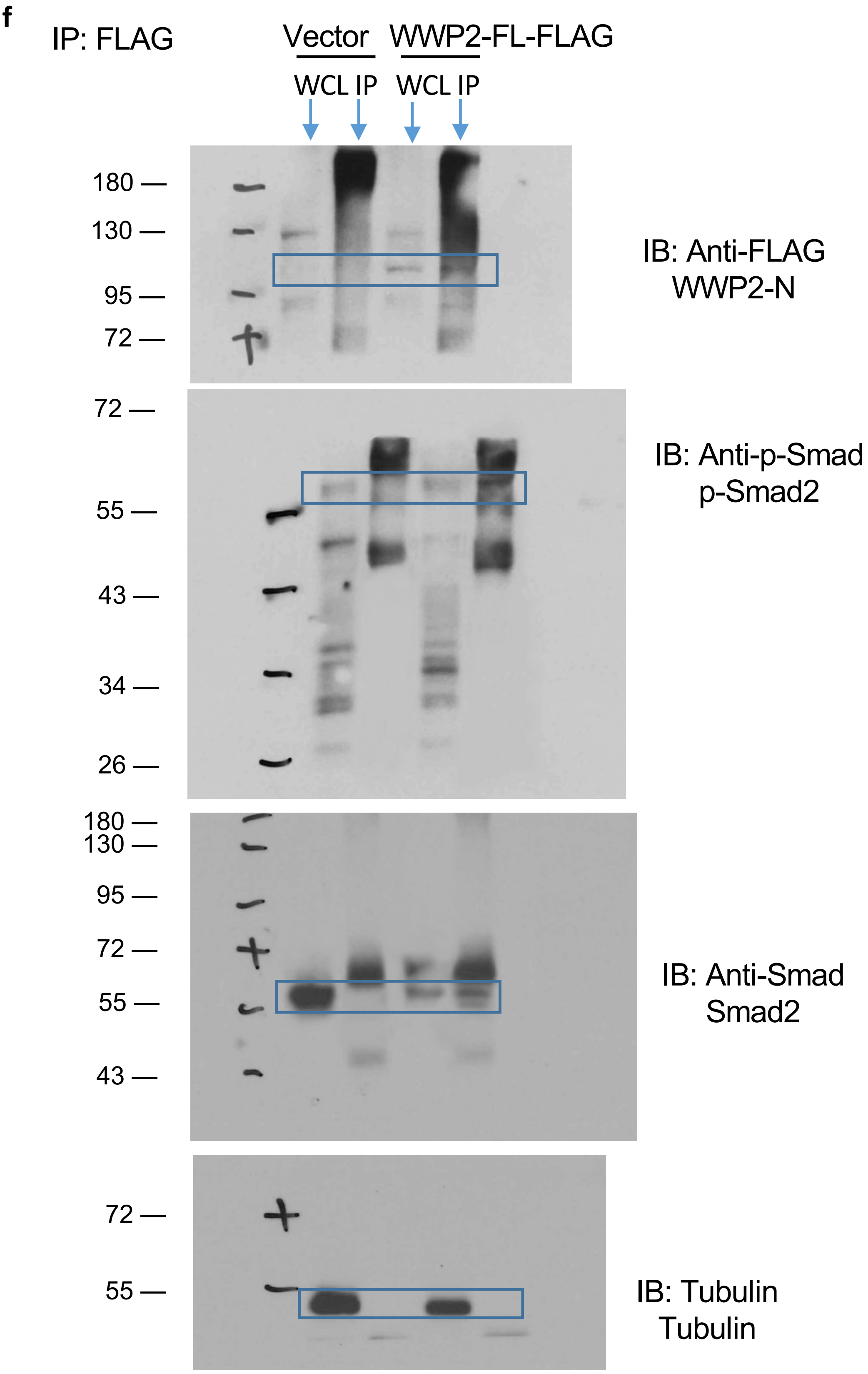

Original blot of Supplementary Figure 11

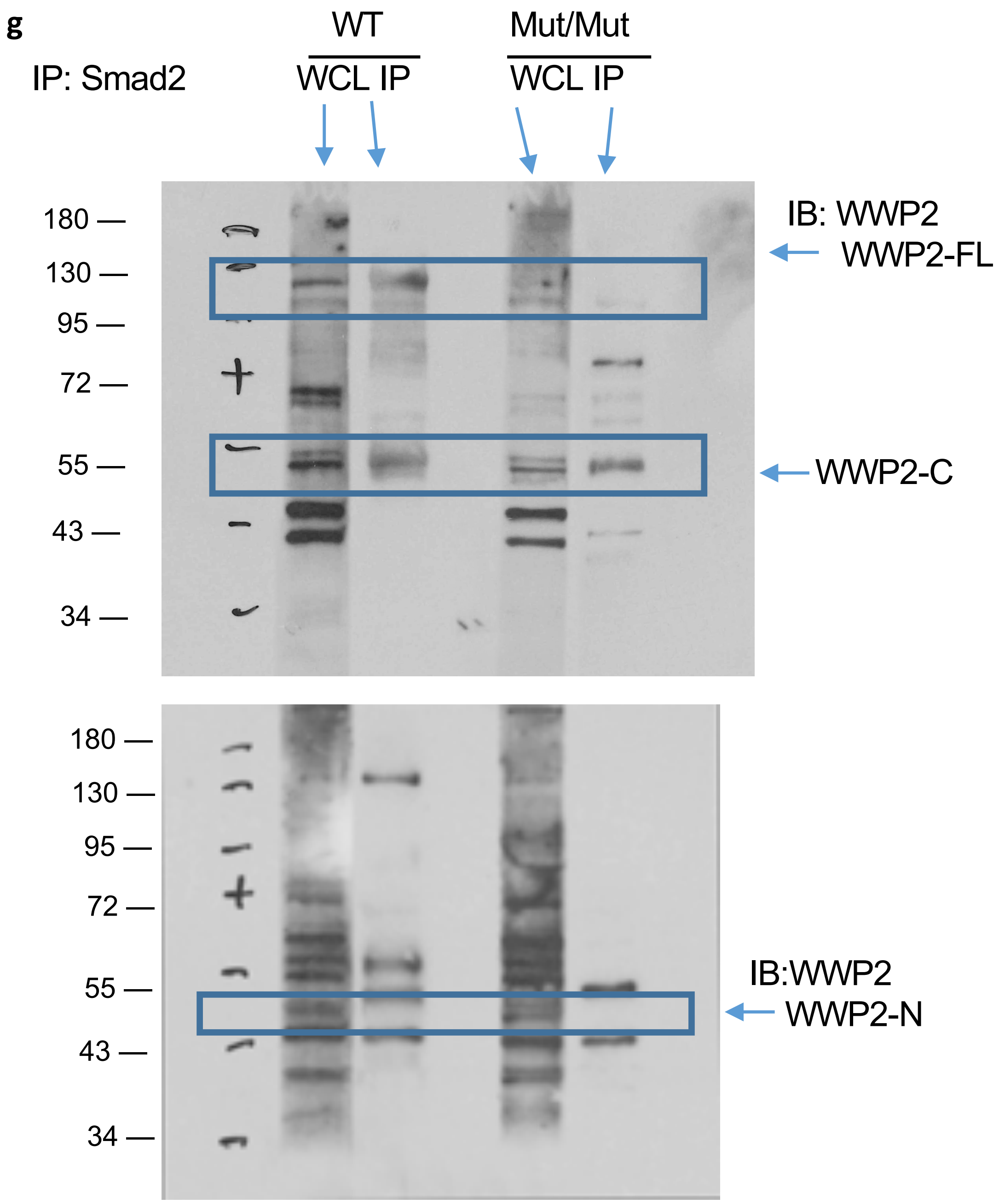

Original blot of Supplementary Figure 11

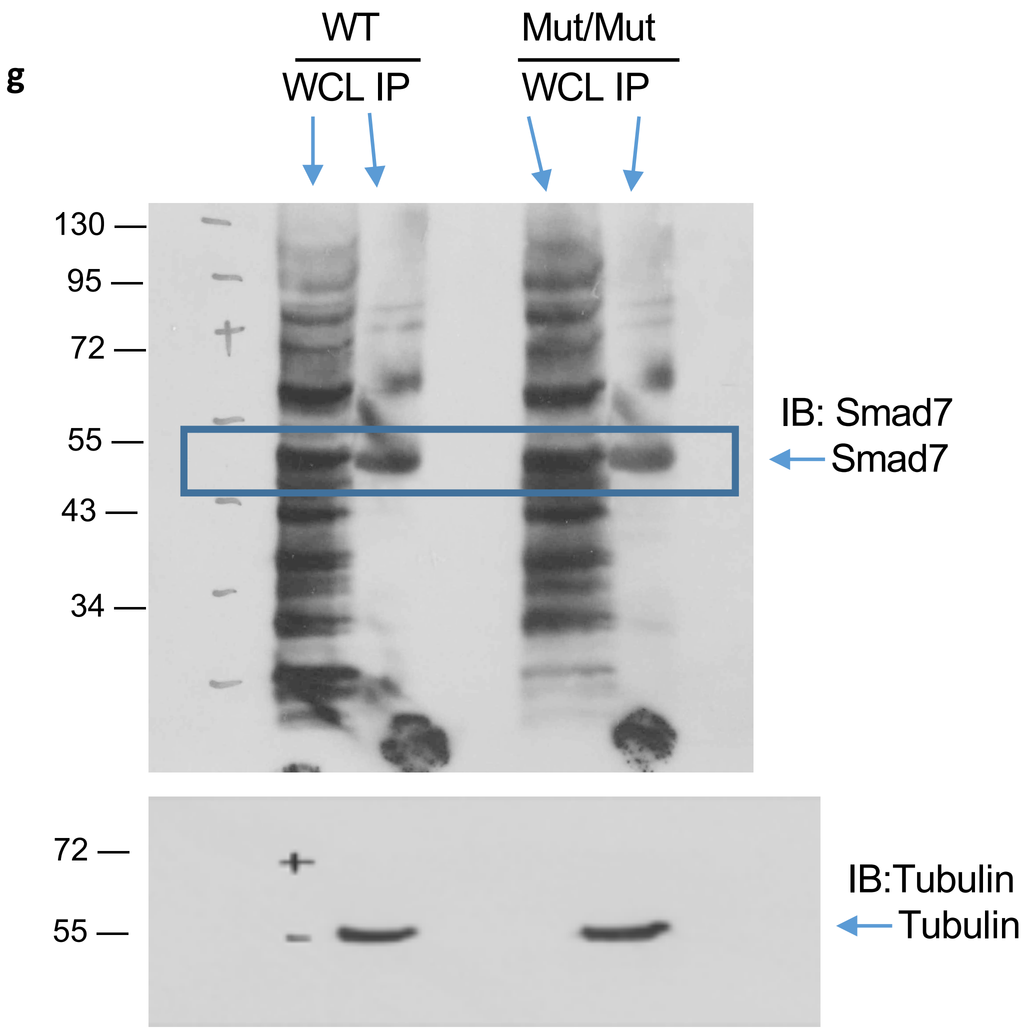

Original blot of Supplementary Figure 11

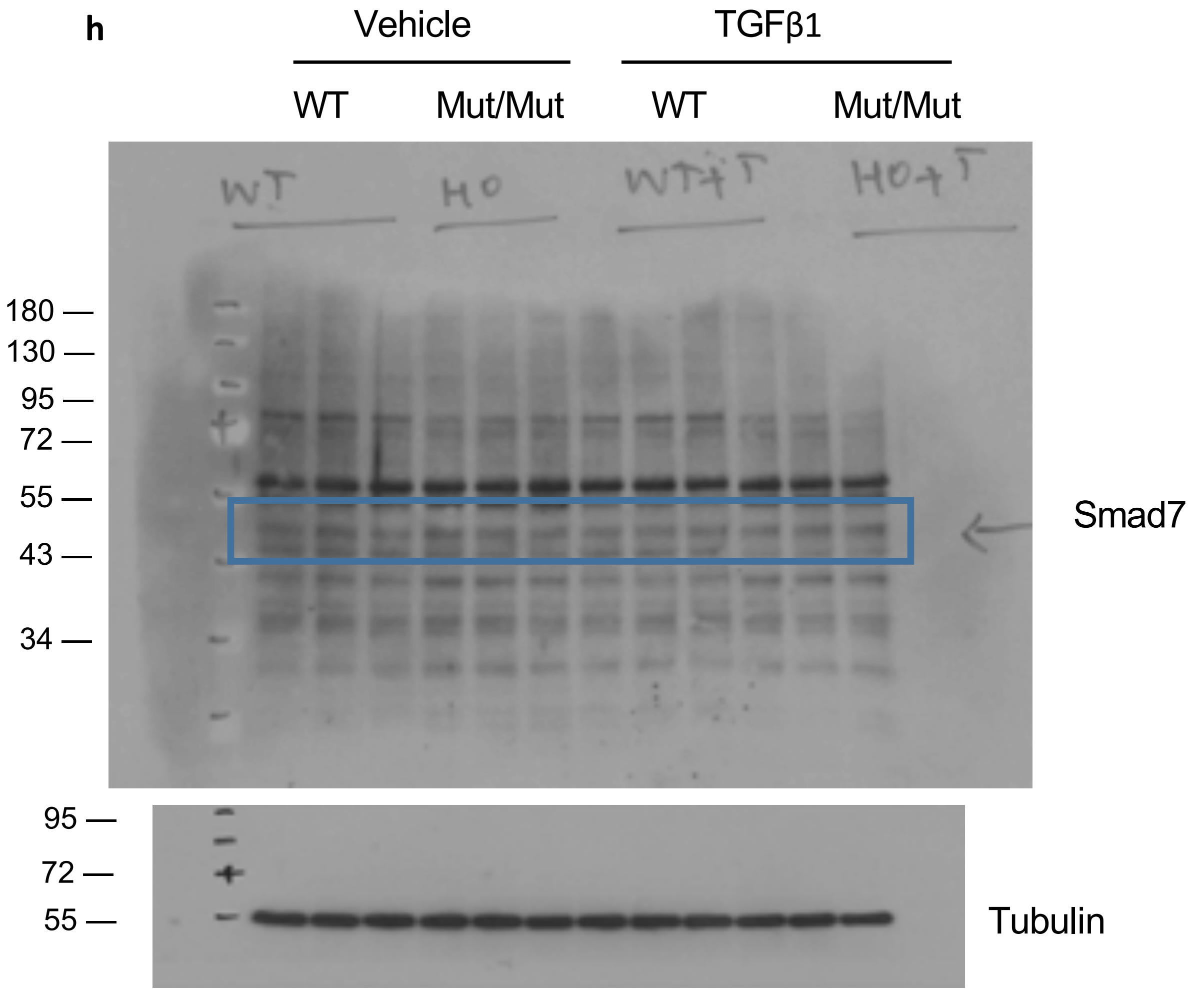

Original blot of Supplementary Figure 11

I

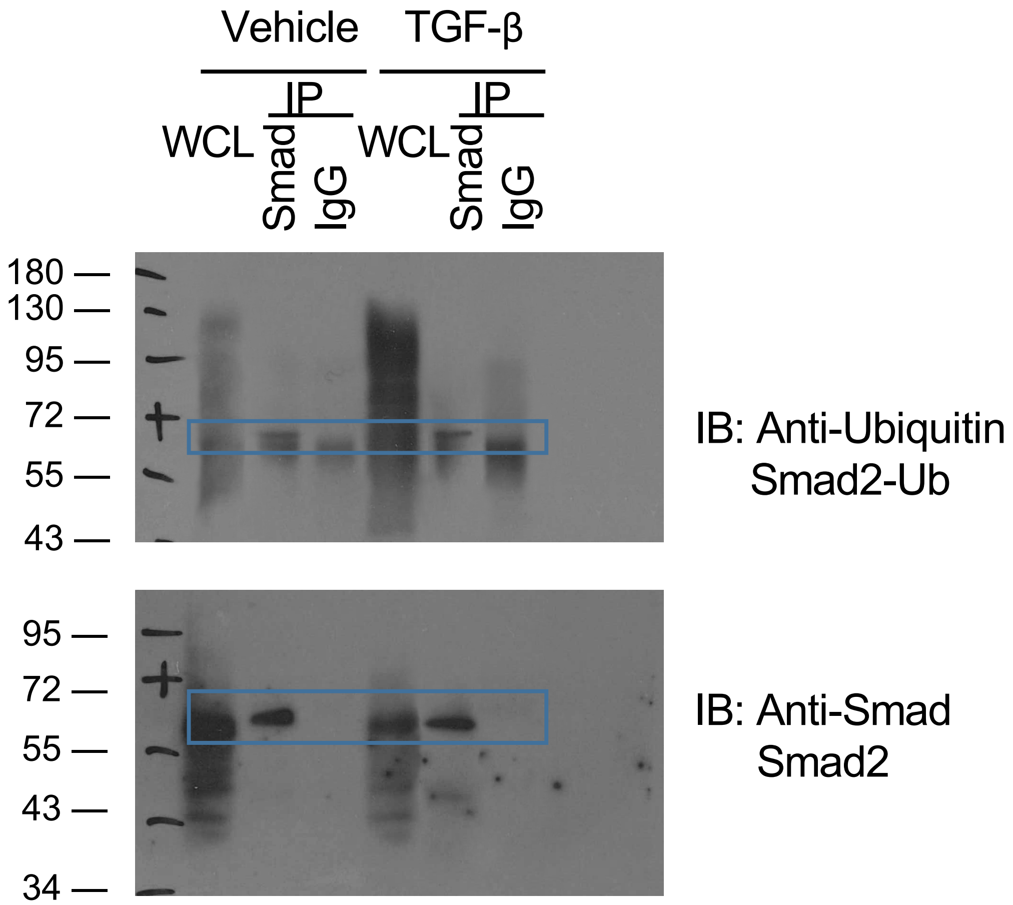

Original blot of Supplementary Figure 12

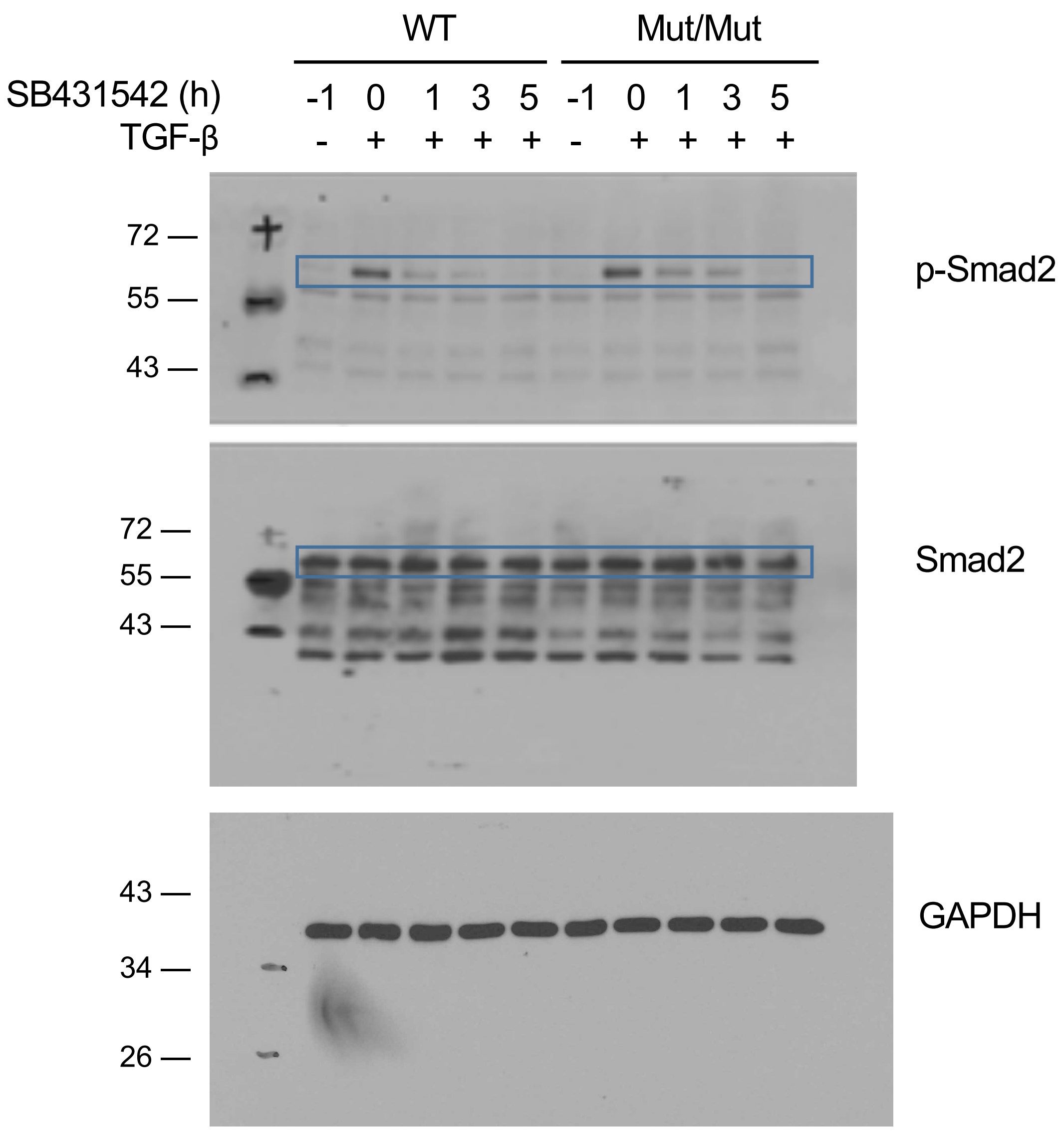

Supplement: Supplementary file 9 — Source Data [file 41467_2019_11551_MOESM9_ESM.pdf]
